# Radiation dosimetry and first therapy results with a ^124^I/^131^I-labeled small molecule (MIP-1095) targeting PSMA for prostate cancer therapy

**DOI:** 10.1007/s00259-014-2713-y

**Published:** 2014-02-28

**Authors:** Christian M. Zechmann, Ali Afshar-Oromieh, Tom Armor, James B. Stubbs, Walter Mier, Boris Hadaschik, John Joyal, Klaus Kopka, Jürgen Debus, John W. Babich, Uwe Haberkorn

**Affiliations:** 1 Department of Nuclear Medicine, University Hospital Heidelberg, Im Neuenheimer Feld 400, 69120 Heidelberg, Germany; 2 Molecular Insight Pharmaceuticals, Boston, MA USA; 3 Radiation Dosimetry Systems RDS, Inc., Apharetta, GA USA; 4 Department of Urology, University Hospital Heidelberg, Heidelberg, Germany; 5 Division Radiopharmaceutical Chemistry, DKFZ, Heidelberg, Germany; 6 Department of Radiation Oncology, University Hospital Heidelberg, Heidelberg, Germany; 7 Clinical Cooperation Unit Nuclear Medicine, DKFZ, Heidelberg, Germany; 8 Division of Radiopharmacy, Department of Radiology, Cornell University, New York, NY USA

**Keywords:** Prostate cancer, PSMA targeting, Radioiodinated PSMA ligand, Biodistribution, Absorbed dose estimates, Dosimetry

## Abstract

**Introduction:**

Since the prostate-specific membrane antigen (PSMA) is frequently over-expressed in prostate cancer (PCa) several PSMA-targeting molecules are under development to detect and treat metastatic castration resistant prostate cancer (mCRPC). We investigated the tissue kinetics of a small molecule inhibitor of PSMA ((*S*)-2-(3-((*S*)-1-carboxy-5-(3-(4-[^124^I]iodophenyl)ureido)pentyl)ureido)pentanedioicacid; MIP-1095) using PET/CT to estimate radiation dosimetry for the potential therapeutic use of ^131^I-MIP-1095 in men with mCRPC. We also report preliminary safety and efficacy of the first 28 consecutive patients treated under a compassionate-use protocol with a single cycle of ^131^I-MIP-1095.

**Methods:**

Sixteen patients with known prostate cancer underwent PET/CT imaging after i.v. administration of ^124^I-MIP-1095 (mean activity: 67.4 MBq). Each patient was scanned using PET/CT up to five times at 1, 4, 24, 48 and 72 h post injection. Volumes of interest were defined for tumor lesions and normal organs at each time point followed by dose calculations using the OLINDA/EXM software. Twenty-eight men with mCRPC were treated with a single cycle of ^131^I-MIP-1095 (mean activity: 4.8 GBq, range 2 to 7.2 GBq) and followed for safety and efficacy. Baseline and follow up examinations included a complete blood count, liver and kidney function tests, and measurement of serum PSA.

**Results:**

I-124-MIP-1095 PET/CT images showed excellent tumor uptake and moderate uptake in liver, proximal intestine and within a few hours post-injection also in the kidneys. High uptake values were observed only in salivary and lacrimal glands. Dosimetry estimates for I-131-MIP-1095 revealed that the highest absorbed doses were delivered to the salivary glands (3.8 mSv/MBq, liver (1.7 mSv/MBq) and kidneys (1.4 mSv/MBq). The absorbed dose calculated for the red marrow was 0.37 mSv/MBq. PSA values decreased by >50 % in 60.7 % of the men treated. Of men with bone pain, 84.6 % showed complete or moderate reduction in pain. Hematological toxicities were mild. Of men treated, 25 % had a transient slight to moderate dry mouth. No adverse effects on renal function were observed.

**Conclusion:**

Based on the biodistribution and dose calculations of the PSMA-targeted small molecule ^124^I-MIP-1095 therapy with the authentic analog ^131^I-MIP-1095 enables a targeted tumor therapy with unprecedented doses delivered to the tumor lesions. Involved lymph node and bone metastases were exposed to estimated absorbed doses upwards of 300 Gy.

**Electronic supplementary material:**

The online version of this article (doi:10.1007/s00259-014-2713-y) contains supplementary material, which is available to authorized users.

## Introduction

Surgery, radiation and cytotoxic drugs are the pillars of treatment of oncological diseases. Generally, surgery and radiation cannot be fundamentally improved in the case of disseminated disease. The efficiency of systemic tumor treatments is limited by the side effects of commonly used standard chemotherapeutics and, thus, the resulting limited amount of the respective standard chemotherapeutic is not sufficient for an effective treatment of most solid tumors.

Once metastasized, prostate cancer becomes one of the most aggressive types of tumors. It affects at least 2 million men in the United States and 4 million men in Europe [1] http://globocan.iarc.fr/factsheets/cancers/prostate2008 [2], and most patients with metastatic castrate resistant prostate cancer (mCRPC) will relapse due to inefficient treatment of the primary lesion. [3]

During the past decade, new therapeutic options, such as new anti-androgen therapies, systemic radiopharmaceuticals, including radium-223 chloride, immunotherapeutics/vaccines and second generation chemotherapeutics such as cabazitaxel, were approved for mCRPC patients. Despite these developments, mCRPC will claim the lives of more than 250,000 men worldwide each year [4]. Alternative therapeutic options for men with mCRPC are urgently needed.

There are two possibilities to extend the therapeutic window required to eradicate aggressive tumors: a) differential action of the drug in the tumor cells and b) specific uptake of the drug. Fortunately, prostate tumors specifically express the prostate-specific membrane antigen (PSMA), an ideal target for imaging and targeted systemic treatment of prostate cancer.

PSMA, also known as folate hydrolase I or glutamate carboxypeptidase II, is a 750-amino acid type II transmembrane glycoprotein primarily expressed in normal human prostate epithelium. Since PSMA is over-expressed by virtually all prostate cancers, and its expression is further increased in poorly differentiated, metastatic and hormone-refractory carcinomas, it is an attractive target for diagnosis and staging of prostate cancer as well as the delivery of endoradiotherapy specifically to prostate cancer cells [5, 6].

Recently, radiolabeled monoclonal antibodies that bind to the extracellular domain of PSMA were shown to accumulate in PSMA-positive prostate tumors in animals. Phase 1 and 2 clinical trials utilizing the PSMA monoclonal antibody J591, radiolabeled with ^90^Y or ^177^Lu have shown promising early results [7–11]. While radiolabeled monoclonal antibodies hold promise for tumor detection and therapy, clinical successes have been limited (with the exception of lymphoma) in large part because these large molecules exhibit poor permeability in solid tumors and slow clearance from the circulation. This combination leads to suboptimal tumor targeting and an increased absorbed dose to red marrow, narrowing the therapeutic window. Low molecular weight compounds, with higher permeability into solid tumors, offer a significant advantage in achieving higher uptake per gram of tumor tissue and a high percentage of specific binding. Furthermore, small molecules display more rapid tissue distribution and faster blood clearance, compared with intact immunoglobulins. These properties often lead to enhanced target to non-target tissue ratios which is important not just for imaging but also for successful application of therapeutic absorbed doses.

Molecular Insight Pharmaceuticals, Inc. has developed a series of ^123^I-labeled and ^99m^Tc-labeled small molecules for targeting the extracellular domain of prostate-specific membrane antigen (PSMA), a target initially selected for the diagnostic assessment of primary and metastatic prostate cancer [12, 13]. The results of the initial clinical investigation of ^123^I-MIP-1072 and ^123^I-MIP-1095 led to the evaluation of these radioiodinated ligands as potential PSMA-targeted radiotherapeutics when radiolabeled with ^131^I [14, 15].

The outstanding tumor selectivity observed with these compounds suggests the possibility of treatment efficacy that may compete with the efficacies attained for other cancers with highly specific drugs such as Gleevec [16]. Different from the application of normal cytotoxic drugs, the possible side effects of endoradiotherapeutics can be estimated by dosimetric calculations for normal tissues.

In preparation for a therapeutic application of ^131^I-MIP-1095 we performed PET/CT measurements with the authentic analog ^124^I-MIP-1095 to obtain dosimetric data for normal organs and assessed safety and efficacy of therapy with ^131^I-MIP-1095 under a compassionate-use protocol in 28 patients with metastatic castrate resistant prostate cancer.

## Methods

### Radiopharmaceuticals

(*S*)-2-(3-((*S*)-1-Carboxy-5-(3-(4-[^131^I]iodophenyl)ureido)pentyl)ureido)pentanedioic acid; ^131^I-MIP-1095

The radioiodinatedcompound was prepared by iododestannylation of the trimethylstannyl precursor (*S*)-di-*tert*-butyl 2-(3-((*S*)-1-*tert*-butoxy-1-oxo-6-(3-(4-(trimethylstannyl)phenyl)ureido)-hexane-2-yl)ureido)pentanedioate, to form (*S*)-2-(3-((*S*)-1-carboxy-5-(3-(4-[^131^I]iodophenyl)ureido)pentyl)ureido)pentanedioic acid. [^131^I]NaI (approx. 7 GBq, GE Healthcare) was reacted with 100 μL of a 250 μg/mL solution of (*S*)-di-tert-butyl 2-(3-((*S*)-1-tert-butoxy-1-oxo-6-(3-(4-(trimethylstannyl)phenyl)ureido)hexane-2-yl)ureido)pentanedioate in ethanol and 50 μL of a freshly prepared solution of 0.15 mL 30 % hydrogen peroxide in 0.85 mL acetic acid. The reaction mixture was diluted after 5 min with 1.5 mL of water and loaded onto a SOLA cartridge. The cartridge was washed with 2 mL of water to remove unreacted radioiodide and inorganic and organic salts and dried by a stream of nitrogen.

The (*S*)-2-(3-((*S*)-1-carboxy-5-(3-(4-[^131^I]iodophenyl)ureido)pentyl)ureido)pentanedioic acid ester was eluted from the dry column using 0.5 mL of neat trifluoroacetic acid (TFA) and deprotected at room temperature for 15 min to cleave the *tert*-butyl ester protecting groups. After the deprotection was complete, the solution was diluted with water (5 mL) and trapped on a Plexa cartridge. The cartridge was washed with 5 mL 20 % ethanol in water and eluted with 1 mL of 100 % ethanol, wherein the solution was passed through a GV sterile filter. The final solution was diluted with 9 mL of sterile water and 200 μL phosphate buffer concentrate to prepare the final formulation matrix containing 10 % ethanol. The radioiodinated compound was analyzed by HPLC on a Chromolith® Performance RP-18e column (100 × 3 mm^2^) using a linear gradient from 0 % to 100 % of acetonitrile in water (both containing 0.1 % TFA) over 5 min. UV absorbance was detected at 214 nm. Radiochemical yields ranged from 38 to 78 %, average radiochemical purity was 97 %, and specific activity ranged from 1.5 to 6.4 mCi/μmol (55.5 to 236.8 MBq/μmol). A slightly modified procedure to that described above was used to prepare ^124^I-MIP-1095. ^124^I was purchased from Eckert & Ziegler (Berlin, Germany).

### Glu-NH-CO-NH-Lys(Ahx)-[^68^Ga]-HBED-CC

The PSMA-binding ligand Glu-NH-CO-NH-Lys(Ahx)-HBED-CC was radiolabeled with gallium-68 and used pre- and post-therapy for assessment of tumor burden in patients undergoing ^131^I-MIP-1095 therapy. ^68^Ga-labeled Glu-NH-CO-NH-Lys(Ahx)-HBED-CC was prepared as previously described [17].

### Dosimetry imaging and pharmacokinetics of ^124^I-MIP-1095

Sixteen patients underwent dosimetry scans as described in the following paragraphs. All patients had histologically proven prostate cancer – either by biopsy or by prostatectomy. The patients had several different treatments in advance of our exam. Eleven men had a radical prostatectomy, two received brachytherapy, all patients received hormone ablation therapy, nine received additional radiotherapy of the prostate and four received radiotherapy elsewhere for their disease, and six had prior chemotherapy. The median PSA value was 110.5 ng/ml at the time of our scans (range 1.13 to 577 ng/dl). The median age was 71.9 years (range 55.2 to 76.6 years). Median Gleason score was 7 (range 6 to 9).

The patients were scheduled for PET/CT using ^124^I-MIP-1095 in order to detect tumor lesions for staging in advance of a possible PSMA-therapy. All patients gave their written informed consent for the examination after extensive education. The evaluation of the data was approved by the local ethics committee (protocol number S-321/2012).

Depending on the efficiency of radiolabeling (range 26 to 105 MBq, supplementary data Table A) the patients received a median activity of 67 MBq (range 26 to 105 MBq) of ^124^I-MIP-1095 intravenously followed by a saline flush with 10 mL of 0.9 % NaCl. The median of the patient weight was 77.5 kg (range 70 to 112.5 kg) resulting in a median activity of 0.87 MBq/kg body weight (range 0.27 to 1.4 MBq/kg bw).

### PET/CT image acquisition

Serial PET/CT images were collected using a Siemens Biograph 6 PET/CT system (Erlangen, Germany) for each patient beginning approximately 1 h post-administration (prior to voiding) and again at approximately 4, 24, 48, 72 and/or 96 h (Supplementary data Table A). A non-contrast-enhanced CT scan was performed 1 h post tracer injection using the following parameters: slice thickness of 5 mm; increments of 0.8 mm; soft tissue reconstruction kernel; 130 keV and 80 mAs. Immediately after CT scanning, a whole body PET was acquired in 3D mode from head to toe in two serial acquisitions at the initial time point and head to mid-thigh at subsequent time points. Only head to mid-thigh acquisitions were obtained at the initial time point in two of 16 patients. For each bed position (16.2 cm, overlapping scale: 4.2 cm) we used 4 min. acquisition time with a 15.5 cm field of view (FOV). Typically five PET time points were obtained for each patient (*n* = 13). In three cases only four (*n* = 1) or three scans (*n* = 2) were available due to technical failure, logistical reasons or claustrophobia of the patient.

The emission data were corrected for randoms, scatter and decay. Reconstruction was conducted with an ordered subset expectation maximization algorithm (OSEM) with four iterations/eight subsets and Gauss-filtered to a transaxial resolution of 4.2 mm at FWHM (full width at half maximum). Attenuation correction was performed using the low dose non-enhanced computed tomography data. PET and CT were performed using the same protocol for every patient on a BIOGRAPH-6 PET/CT scanner (Siemens, Germany). No additional manufacturer-supplied corrections for prompt gamma coincidences were applied at the time of acquisition. Axial slices were reconstructed with CT-based measured attenuation correction and iterative ordered subsets expectations maximization (OSEM) algorithm.

Calibration of the PET activity was performed by imaging a volume of ^124^I measured in an ISOMED 1010 dose calibrator (Dresden, Germany) to develop a calibration factor which allows for correction of prompt gamma emissions and the low positron fraction of ^124^I. We measured the activity of a 20 mL imaging standard containing a minimal amount of ^124^I-MIP-1095 in advance of each PET/CT scan in the dose calibrator and set this syringe adjacent to the head of the patient during the scan.

### Image quantification

Volumes of interest (VOIs) were created from the sum of adjacent transaxial regions of interest (ROIs) manually drawn along the boundaries of a standard set of normal organs and tissues, using CT as a guide, including salivary glands, brain, heart, kidneys, liver, lungs, spleen, thyroid, muscle, abdomen, tumor lesions and whole body. VOIs were created on the 4 h time point and copied to all other time points for each patient and repositioned if needed using the fused CT for proper placement. Count activity for each volume was extracted using a commercially available nuclear medicine workstation (Hermes Medical Systems, Stockholm, Sweden).

Initial whole body count activity from head and mid-thigh was summed with the mid-thigh to toe counts to determine total body uptake immediately post-injection (1 h p.i. and prior to voiding) and considered to be equal to 100 % of the injected activity of ^124^I-MIP-1095. In two patients where leg count data were not available, the initial head to mid-thigh VOI count data were used leading to an increase in the calculated percent of the injected activity in organ VOIs. Hence, for two patients, the organ absorbed doses are biased slightly higher.

All organ (and whole body) VOI data were divided by the initial total body VOI value to obtain the fractional amount of injected activity in each VOI/organ and corrected for radioactive decay of ^124^I to the time of injection. The resulting decay corrected estimates of factional activity distribution were used in the biomodeling.

### Source organ time-activity curve fitting (biomodeling)

Data describing the fraction of the injected activity in each source organ were mathematically simulated. For this data set, bi-exponential functions were iteratively fit to each source organ time-activity curve using a nonlinear least-squares regression algorithm (SAAM II v1.2 software; The SAAM Institute, Inc., Seattle, Washington.www.saam.com). The form of the bi-exponential equation, *A(t)*, is given below:

$$ A(t)={a}_1{e}^{-{\lambda}_1t}+{a}_2{e}^{-{\lambda}_2t} $$

Where *a*_i_ is the i^th^ fractional uptake and *λ*_i_ is its associated biological removal rate (h^-1^). The residence time, τ, is obtained by analytically integrating the curve-fit equation, A(t) from *t* = 0 to infinity, after multiplying each term by the physical decay term (i.e., ${e}^{-{\lambda}_P\;t}$). The effective removal rate is the sum of the biological removal rate and the physical decay rate of the nuclide. One patient had three images and hence a set of mono-exponentials was used in that patient’s biomodeling.

The source organ residence times were calculated for ^124^I and ^131^I, as described above, using each radionuclide’s respective physical half-life. Implicit is the assumption that changing radioiodine from isotope ^124^I to isotope ^131^I does not alter the compound’s biodistribution or kinetics.

### Excretory clearance and total body residence times

Urinary clearance was estimated from the whole body image data. The whole body time-activity curve was fit to a bi-exponential function. The whole (total) body residence time was calculated by integrating the whole body time activity curve fit equation, after subtracting the estimated fecal excretion fraction from the slower clearing component of the whole body time activity curve. Integration of this “corrected” whole body retention function yielded the corrected whole body residence time. The parameters (fraction and associated biological removal half-time) of the corrected whole body time activity curve were used as input data for the dynamic (urinary) bladder model. The urinary bladder contents’ residence times were calculated using the OLINDA implementation of Cloutier’s dynamic bladder model assuming a 4.8 h void schedule.

The remainder of the body residence time was calculated as the corrected total body residence time minus all other residence times, except urinary bladder and intestines. In this report, the gastrointestinal tract comprised the small bowel, upper large intestine (proximal colon) and lower large intestine (distal colon).

### Dosimetry

#### Absorbed dose calculations

The OLINDA/EXM software was used to estimate the absorbed doses [18]. The OLINDA/EXM software has a complete series of dosimetry phantoms corresponding to different age “Reference Human” bodies. In this work, the adult male dosimetry phantom was used exclusively for computing all absorbed dose estimates. The urinary bladder was assumed to void regularly at 4.8-h intervals and the gut transit times of the human adult male were assumed. Kidney absorbed doses were calculated using the adult male phantom’s default kidney mass.

The calculation of absorbed dose to salivary glands was performed using spherical S-values. The S-value is the absorbed dose per unit cumulated activity and varies inversely with mass. A table of such S-values [mGy/(MBq-s)] was calculated from OLINDA/EXM. The S-value for the salivary gland (77 g) was interpolated from these data. Since the salivary gland was considered to be a single sphere of 77 g, the salivary absorbed doses are necessarily conservative. Further details are given in Table 1.

**Table 1:** Summary of absorbed dose estimates for ^124^I-PSMA and ^131^I-PSMA targeted small molecule

	Mean	SD	Min	Max	Mean	SD	Min	Max
Target Organ	mSv/MBq	mSv/MBq	mSv/MBq	mSv/MBq	mSv/MBq	mSv/MBq	mSv/MBq	mSv/MBq
Salivary Glands	3.76	2.29	0.28	9.07	4.62	3.10	0.28	11.04
Liver	1.66	0.61	0.66	2.90	1.47	0.70	0.27	2.51
Kidneys	1.39	0.56	0.39	2.43	1.45	0.68	0.50	3.25
LLI Wall	0.98	0.26	0.60	1.50	1.32	1.72	0.18	6.56
Thyroid	0.93	0.99	0.20	3.94	0.91	0.25	0.53	1.39
Spleen	0.77	0.32	0.43	1.65	0.69	0.34	0.32	1.63
ULI Wall	0.70	0.15	0.47	0.95	0.65	0.29	0.28	1.14
Gallbladder Wall	0.69	0.11	0.51	0.91	0.58	0.30	0.23	1.41
Heart Wall	0.61	0.24	0.35	1.16	0.58	0.15	0.33	0.84
UrinaryBladder Wall	0.57	0.10	0.34	0.68	0.51	0.11	0.25	0.64
Adrenals	0.57	0.10	0.43	0.77	0.49	0.13	0.32	0.79
Pancreas	0.56	0.11	0.41	0.78	0.44	0.15	0.24	0.72
Small Intestine	0.52	0.12	0.33	0.71	0.43	0.14	0.25	0.71
Osteogenic Cells	0.51	0.15	0.30	0.74	0.42	0.15	0.21	0.66
Ovaries	0.48	0.12	0.29	0.67	0.42	0.23	0.18	1.11
Lungs	0.47	0.17	0.27	0.90	0.39	0.16	0.18	0.64
Uterus	0.46	0.12	0.29	0.64	0.38	0.16	0.18	0.65
Stomach Wall	0.46	0.11	0.30	0.65	0.38	0.15	0.18	0.62
Thymus	0.38	0.11	0.23	0.56	0.34	0.14	0.16	0.57
Muscle	0.37	0.10	0.23	0.52	0.32	0.14	0.15	0.55
Red Marrow	0.36	0.09	0.23	0.51	0.31	0.14	0.13	0.56
Testes	0.34	0.11	0.19	0.51	0.29	0.12	0.14	0.49
Breasts	0.31	0.08	0.19	0.45	0.29	0.12	0.13	0.48
Skin	0.28	0.08	0.17	0.39	0.27	0.12	0.12	0.47
Brain	0.13	0.03	0.07	0.18	0.10	0.03	0.04	0.15
Total Body	0.42	0.09	0.28	0.58	0.37	0.13	0.20	0.62
Effective Dose Equivalent (mSv/MBq)	0.65	0.12	0.47	0.89	0.60	0.15	0.41	0.99
Effective Dose (mSv/MBq)	0.58	0.11	0.41	0.78	0.54	0.14	0.35	0.84

*ULI wall* upper large intestinal wall. *LLI wall* lower large intestinal wall ^124^I-PSMA ^131^I-PSMA

#### Therapy with ^131^I-MIP-1095

Between July 2011 and June 2012, 28 men with mCRPC who demonstrated PSMA-avid lesions on imaging received a single therapeutic activity of ^131^I-MIP-1095 (mean activity: 4.8 GBq, range 2.0–7.2 GBq). Post administration, patients were treated as in-patients on the Nuclear Medicine therapy ward for 5–7 days according to German radiation protection laws.

Blood samples were collected from all patients for the measurement of hematology parameters, GOT, GPT, GGT, CHE, AP, bilirubin, serum PSA, sodium, potassium, calcium, phosphate, and thyroid parameters (fT3, fT4, TSH). Thirty minutes prior to therapy 60 drops of sodium perchlorate (Irenat®, Bayer, Berlin, Germany) were given. Prior to therapy the patients received 1,000 mL of 0,9 % NaCl solution over 1 day (if medically indicated a second NaCl infusion of 1,000 ml was given). The therapy solution was administered by intravenous infusion over 20 min. In order to reduce therapy induced damage of the salivary glands, the patients received lemon juice and ice packs over the parotids and submandibular glands. On the day of administration, a further 20 drops of sodium perchlorate was given at noon and again in the evening. In total, the patients received 60 drops per day (20 × 3) of sodium perchlorate as well as lemon juice and ice packs to reduce organ perfusion. A first set of hematology parameters, liver and kidney values and electrolytes was acquired on day 3–5 after therapy administration. Furthermore, hematology parameters, liver and kidney values, electrolytes and serum PSA were determined on the day of discharge from the hospital (day 7). Whole body scintigraphy was acquired on day 6 to 11 (median 7 days, range 6 to 11), and in one case also at 17 days p.i. The patients were then followed further for hematology parameters including creatinine, BUN and serum PSA values. The patient characteristics including previous therapies are given in supplementary data Table B.

## Results

### Radiopharmaceuticals

The labeling of MIP was performed by radioiododestannylation of the trimethyltin precursor with either ^124^I for imaging or ^131^I for therapy, respectively, using hydrogen peroxide as the oxidant. Interestingly, no significant dependence of the yields obtained from the amount of iodide used was observed. The no-carrier-added radioiodinated PSMA ligands were purified by solid phase extraction and obtained with high radiochemical yields after isolation and excellent purities > 97 %. High specific activity ranging from 1.5 to 6.4 mCi/μmol (55.5 to 236.8 MBq /μmol) could be obtained. Co-injections of the radiolabeled material with an independently prepared iodinated standard confirmed the identity of the radioiodinated compounds.

### Imaging and pharmacokinetics of ^124^I-MIP-1095

**Fig. 1:**
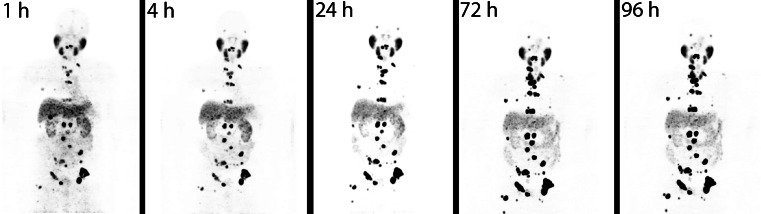
^124^I-MIP-1095 PET images (maximal intensity projection) of patient 01 as a function of time

**Fig. 2:**
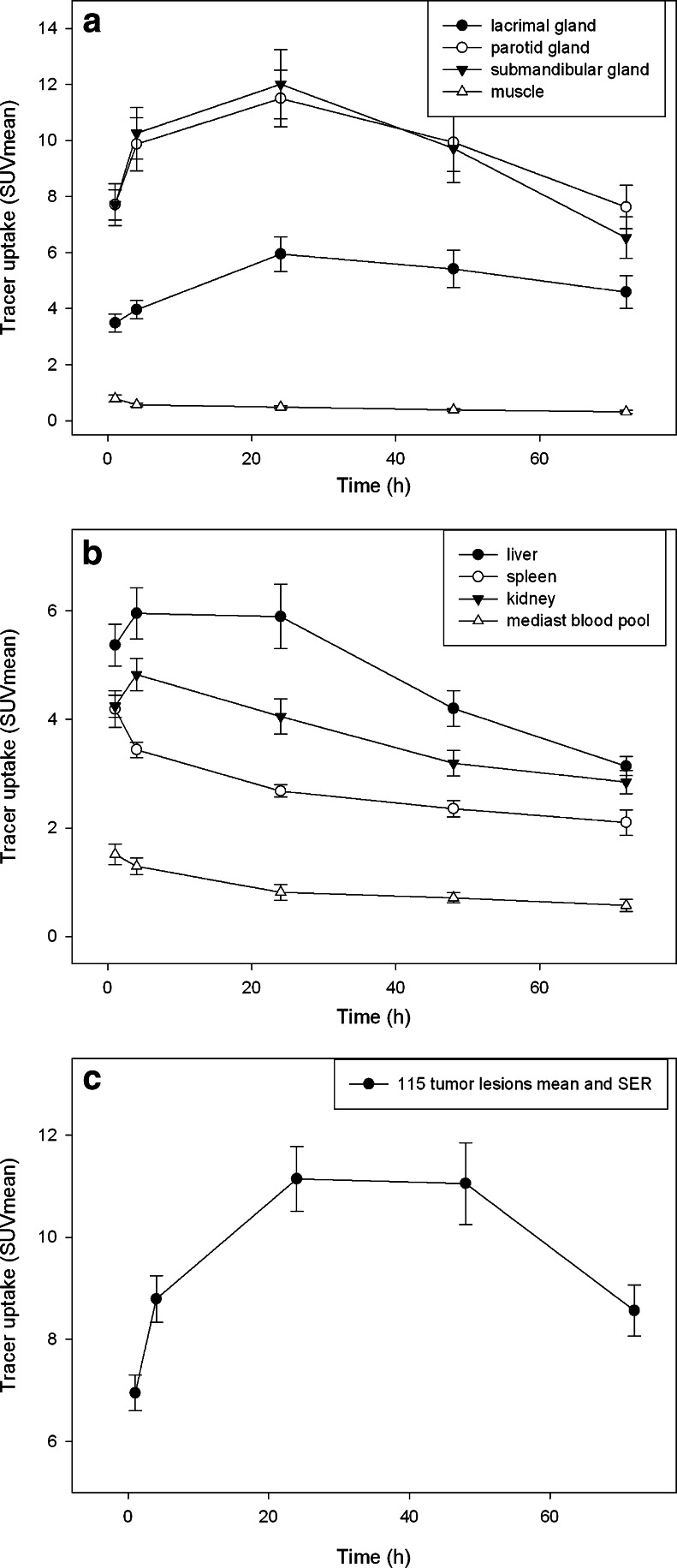
Average SUVs for normal organs (**a** and **b**) and tumor (**c**). Mean tumor SUVs are determined from 110 individual lesions in 16 patients

In addition to the excellent uptake in the prostate tumors and their metastases, maximum intensity projections of the PET/CT scans showed high uptake in the salivary glands, often pronounced in the parotids (Figs. 1 and 2). Focal uptake was also seen in the lacrimal glands and moderate uptake observed in the liver and proximal intestine (duodenum and proximal jejunum). Depending on the time of imaging, the excretion of the radioactive compound led to visually detectable activity in the kidneys and the bladder. The uptake in tumor lesions peaked during the first 24 h after tracer administration. In both bone and soft tissue tumor lesions the distribution stabilizes at a high level and only slowly decreases over time (Fig. 2, Supplementary data Table C).

### Dosimetry

The absorbed dose estimates for ^124^I-MIP-1095 and ^131^I-MIP-1095 are listed for each organ in Table 1, and take into account the voiding time as well as the gut transit time. The organs receiving the highest absorbed doses following administration of ^124^I-MIP-1095 are the salivary glands with a mean absorbed dose of 3.8 mSv/MBq, followed by the liver with 1.7 mSv/MBq and the kidneys with 1.4 mSv/MBq.

This leads to an estimated organ absorbed dose for the administered activities (between 26 and 105 MBq of ^124^I-MIP-1095) for the salivary glands of 98–395 mSv. Liver absorbed doses fall in the range of 43–174 mSv. The kidneys received an absorbed dose between 36 and 146 mSv. For estimation of the whole body absorbed dose a mean of 0.42 mSv/MBq was calculated resulting in 11 to 44 mSv based on our range of injected activities. The effective dose of 0.59 mSv/MBq accounts for 15 to 62 mSv.

Based on the biodistribution data obtained from the ^124^I-MIP-1095 PET images, we calculated the absorbed dose for ^131^I-MIP-1095 using the physical decay characteristics of ^131^I. Based on this extrapolation and the biomodeling obtained from the ^124^I-MIP-1095 PET images, the organs receiving the highest absorbed doses following administration of ^131^I-MIP-1095 are the salivary glands (mean dose 4.6 mSv/MBq, followed by the liver (1.5 mSv/MBq) and the kidneys (1.5 mSv/MBq).

This leads to an estimated absorbed dose for the injected therapy activities (mean dose: 4.8 GBq, range 2.0–7.2 GBq) for the salivary glands of 9.2 to 33.3 Sv. Liver radiation doses fall in the range of 2.9 to 10.6 Sv. The kidneys received a total absorbed dose between 2.9 and 10.4 Sv. The mean, total whole body absorbed dose was 0.38 mSv/MBq resulting in 0.76 to 2.7 Sv based on our injected activities.

These data were compared to the dosimetric values reported for the ^90^Y- and ^177^Lu-labeled antibody J591 (Supplementary data Table D) [9, 10]. The ratio ^131^I-MIP-1095 to ^90^Y-J591 or ^177^Lu-J591 showed that the absorbed doses for ^131^I-MIP-1095 were markedly lower than for the ^90^Y-labeled antibody. Compared to the ^177^Lu-labeled antibody the effective dose obtained with ^131^I-MIP-1095 is higher, red marrow and kidney doses are similar and the liver dose is lower.

### Therapy with ^131^I-MIP-1095

**Fig. 3:**
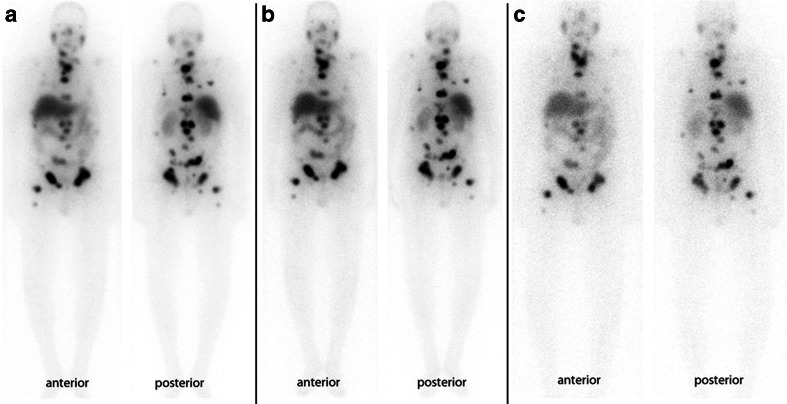
Anterior and posterior whole body scintigrams of ^131^I-MIP-1095 in patient 01 at 7, 10 and 17 days post injection

We treated 28 patients with ^131^I-MIP-1095. Three patients were lost to follow up. Serum PSA values were available for 25 patients and hematologic data for 24 patients. In all patients the tracer was taken up avidly in tumor lesions and remained in these lesions for a prolonged period of time. Figure 3 shows an example where the lesions were visible even 17 days after administration of ^131^I-MIP-1095.

### Side effects and toxicity

A summary of the available medical history, demographics, treatment history, imaging studies, and ^131^I-MIP-1095 treatment activities, and general comments can be found in supplementary data Table B.

**Fig. 4:**
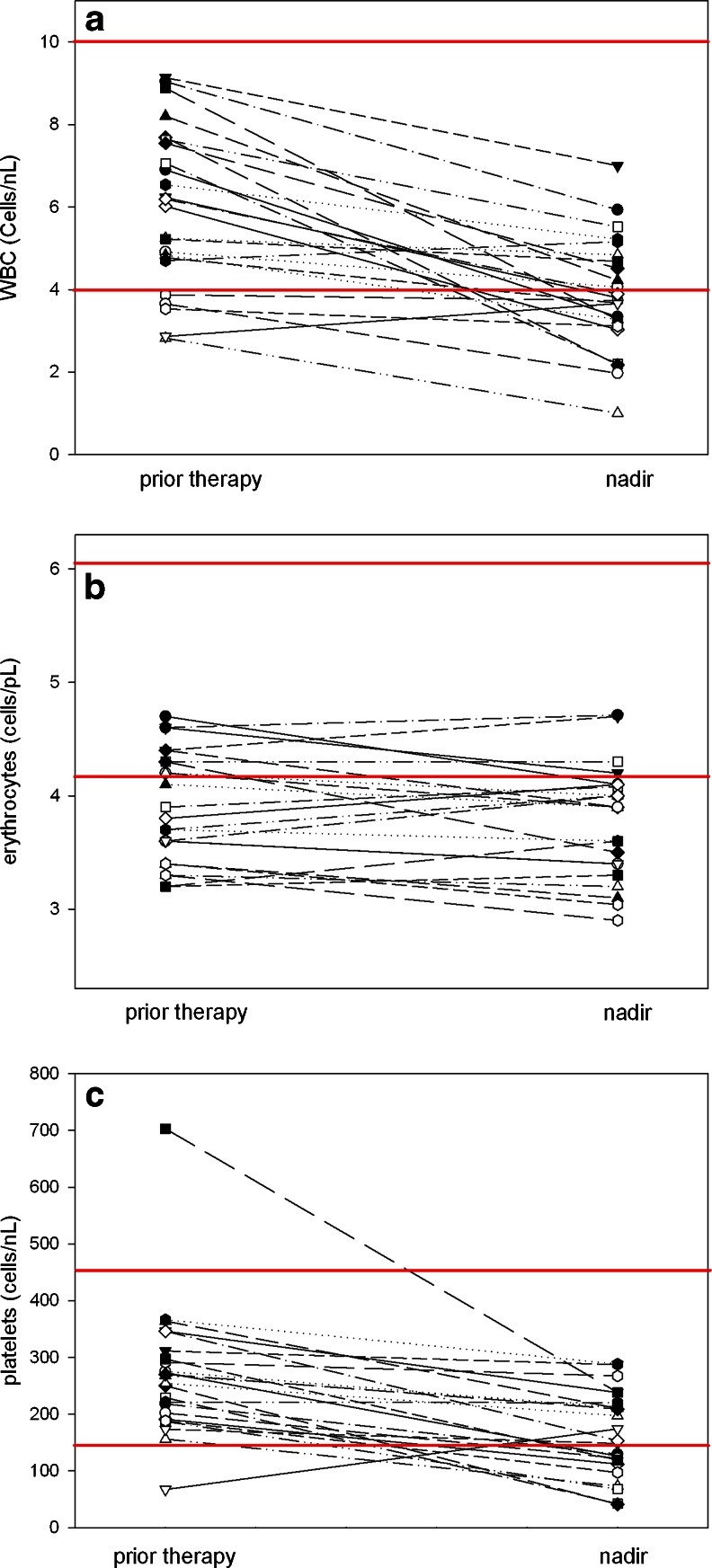
Hematological data for all patients prior to and after treatment; leukocytes (**a**), erythrocytes (**b**) and platelets (**c**). Solid red lines indicate range or normal limit

In 14 patients WBC counts fell below the normal range after therapy (10 patients with grade 1, 3 with grade 2 and one with grade 3 toxicity). However, five of these 14 patients had levels below normal prior to therapy (four grade 1, one with grade 2). Erythrocytes counts fell below the normal range at nadir in 21 patients with 17 patients having lower values prior to therapy. With respect to platelets, 11 patients had a reduction in counts below normal after therapy (eight grade 1, one grade 2 and two grade 3), one having had a value below normal (grade 2) prior to therapy (Fig. 4). The changes in hematological parameters were not related to the activity administered (Supplementary Table E).

**Fig. 5:**
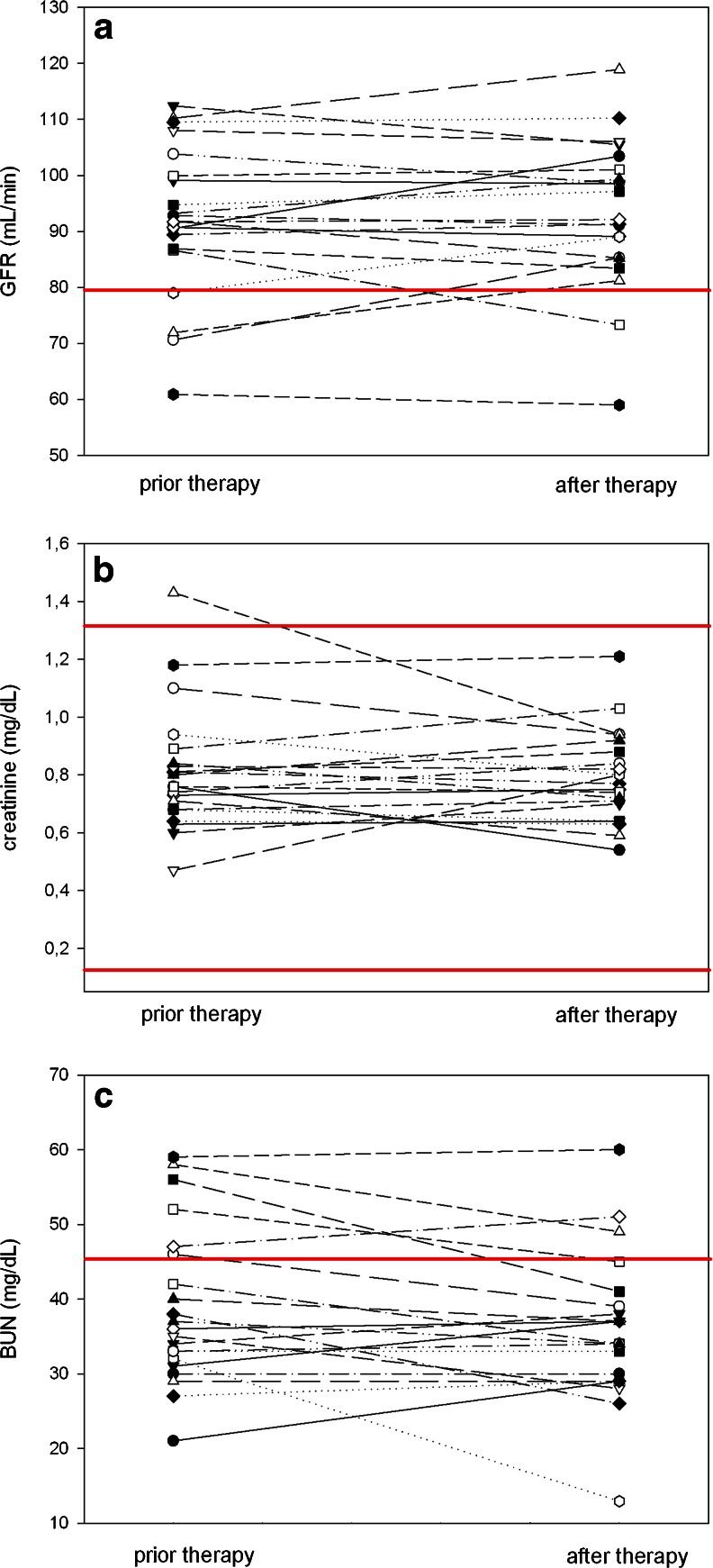
Renal function tests for all patients prior to and after therapy; GFR, measured with ^99m^Tc-MAG_3_ scintigraphy (**a**), Serum creatinine (**b**), Blood urea nitrogen (**c**). Solid red lines indicate range or normal limit

Because of physiological PSMA expression in the kidneys there is concern regarding the potential for radiation toxicity to the kidneys as a possible chronic long-term side effect of PSMA targeted endoradiotherapies. Available clinical data were collected to assess renal function. While the data are inherently limited in terms of long-term follow-up, as the post-treatment interval of most of these patients provides less than a year of follow-up, there is no apparent evidence or negative trend in either calculated GFR or serum creatinine levels. The parameters describing kidney function were largely unchanged: GFR decreased below normal in one patient, improved from below normal to within the normal range in two patients and remained stable below normal in one patient (Fig. 5a). All creatinine levels were in the normal range prior to treatment and remained in the normal range post treatment (Fig. 5b). BUN values were largely unchanged with three patients above normal values after therapy (grade 1) and six patients (grade 1) prior to therapy (Fig. 5c). These findings are consistent with the estimated absorbed doses to the kidney which were significantly below 23 Gy. The evaluation of liver function tests and thyroid hormone revealed no change of these parameters after therapy (data not shown).

In some patients, evidence of non-hematological transient side effects was found: seven patients reported having a slight to moderate xerostomia, and in one patient mucositis was detected. All reported side effects recovered after 3 to 4 weeks. This latter finding is likely due to the high level of radiopharmaceutical accumulation in these organs and the estimated absorbed doses.

### Response

All 28 patients had progressive metastatic hormone refractory disease by serum PSA, radiographic or clinical measures. Thirteen patients complained of bone pain related to their metastases prior to treatment with ^131^I-MIP-1095. After treatment, three out of 13 (23.1 %) patients had reported complete resolution of bone pain and eight (61.5 %) reported a decrease in pain severity. In the remaining two patients, the outcome is unknown.

**Fig. 6:**
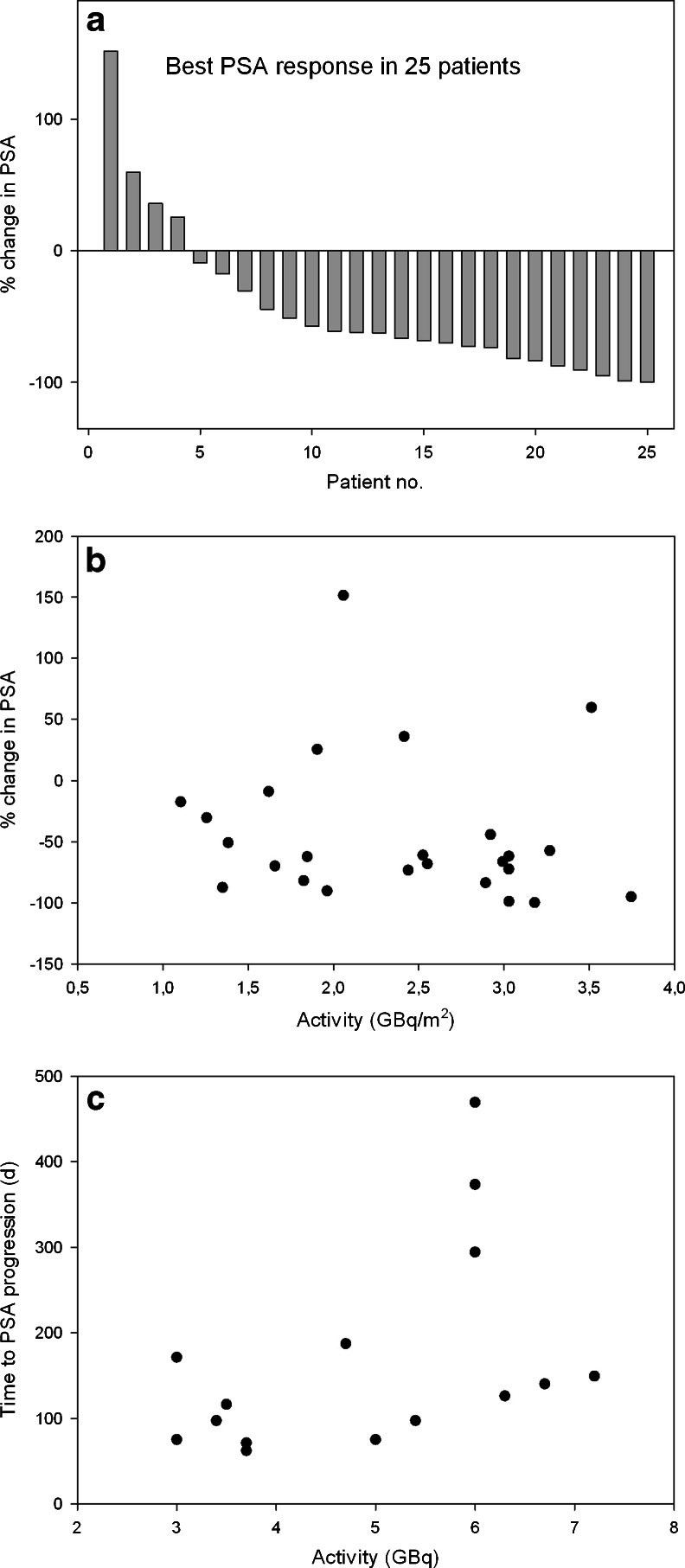
Effects of PSMA ligand therapy on PSA values. **a** Waterfall plot of best PSA response, compared to baseline, after a single cycle of ^131^I-MIP-1095; **b** Relation of change in PSA to the activity given and **c** Relation of the time to PSA progression to the activity given

**Fig. 7:**
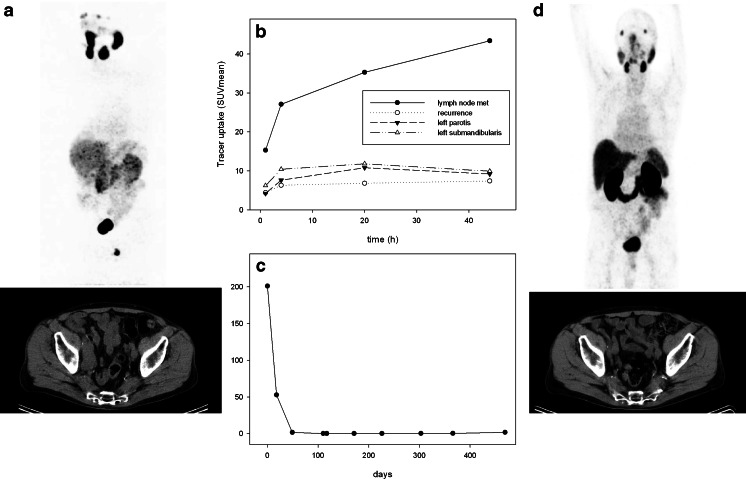
Patient 04. **a** Pre-therapy ^124^I-MIP-1095 PET scans (*top*) and CT scan (*bottom*). **b** Pre-therapy SUV values for LN metastasis, recurrent tumor, submandibular salivary glands and parotid glands as a function of time. **c** Serum PSA values as a function of time from treatment. **d** Post-therapy Glu-NH-CO-NH-Lys(Ahx)-[^68^Ga]-HBED-CC PET scan (*top*) and CT scan (*bottom*)

**Fig. 8:**
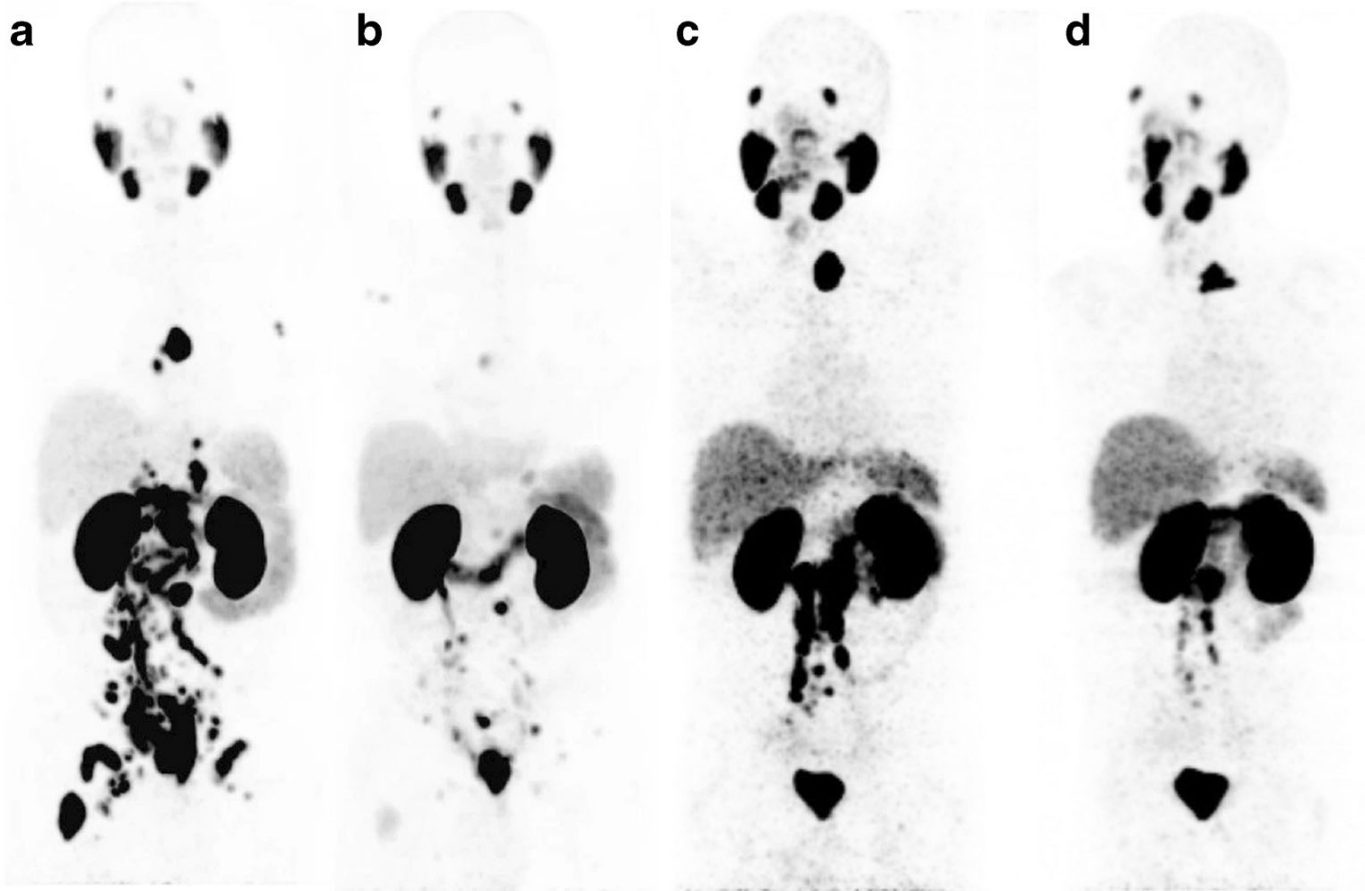
Two patients (**a**, **b** and **c**, **d**) prior (**a**, **c**) and after (**b**, **d**) therapy showing reduced tracer accumulation in their tumor lesions after treatment

Overall best PSA response after a single cycle of ^131^I-MIP-1095 is provided in Fig. 6a. In 60.7 % of patients, a decline in serum PSA levels of ≥50 % was observed; seven (25 %) had more than a 75 % drop in PSA, 10 (35.7 %) had a drop between 50 and 75 %, two (7.1 %) between 25 % and 50 % and two (7.1 %) between 0 and 25 % (Fig. 6). One patient showed a long lasting complete response by serum PSA value and by radiographic imaging (Fig. 7). In four patients, an increase of PSA was observed. As with the hematological parameters, also the changes in PSA value were not related to the activity administered (Fig. 6b). In the 19 patients showing a more than 25 % decrease in PSA the median time to PSA progression was 126 days (range 62 to 469 days; Fig. 6c). A decrease in PSA was associated with a decrease in number and/or intensity of the lesions visualized on the post-therapeutic PET/CT scan with ^68^Ga-labeled Glu-NH-CO-NH-Lys(Ahx)-HBED-CC. Figure 8 shows two patients with a dramatic reduction of tracer uptake in the tumor lesions after one cycle of PSMA treatment.

## Discussion

PET imaging with ^124^I-MIP-1095 showed rapid uptake and long lasting accumulation in prostate cancer metastases with significantly lower uptake in most normal tissues except for parotid and salivary glands, leading to high tumor contrast (Figs. 1 and 2). We have shown that ^131^I-MIP-1095 displays rapid and high uptake and prolonged retention in tumor lesions and that ^124^I-MIP-1095 can be used to confirm PSMA-avidity and estimate normal organ dosimetry prior to treatment. These findings are consistent with previous reports of tumor uptake and retention using ^123^I-MIP-1095 in men with mCRPC [19] and extend our understanding of the tumor and normal tissue kinetics due to the longer half-lives of the radioiodine isotopes ^124^I and ^131^I. This distribution pattern of the tracer fits the PSMA expression profile previously reported for prostate cancer metastases and normal tissues [20–22].

The main elimination process is the renal system, although uptake by the salivary glands and the intestines might suggest also gastrointestinal action. However, we found that the parotids showed a prolonged retention of the radioiodinated PSMA-targeted ligands that even led, in some cases, to an intensified uptake after about 20 h (Fig. 2). This suggests that a forced excretion might not show an effect on the uptake in the salivary glands. The uptake in the proximal small intestine is explained by natural occurrence of PSMA in this organ [21].

Calculations of mean radiation absorbed doses for ^131^I-MIP-1095 based on the pharmacokinetic data derived from the ^124^I-MIP-1095 PET scan images indicate that the normal tissues with the highest absorbed doses are salivary glands, liver, kidney and lower large intestine, respectively (Table 1). A comparison of the dosimetric values for ^131^I-MIP-1095 with the data published for the ^90^Y- and ^177^Lu-labeled J591 antibody [9, 10] revealed favorable results for the small molecule when compared to the ^90^Y-labeled antibody. A comparison with the ^177^Lu-labeled antibody revealed a higher effective dose, a lower liver dose and similar values for bone marrow and kidneys.

According to the dosimetric data salivary glands, liver, kidney and lower large intestine represent potential concern for side effects. Since many endoradiotherapeutic procedures are associated with hematological toxicity we also evaluated the effects of therapy on leucocytes, erythrocytes and platelets. We observed changes in hematological profiles in most patients after therapy, albeit many of these individuals had subnormal values at baseline. This is most probably due to previous systemic therapy affecting the bone marrow, which can be expected for the present patient population. Almost all of these patients had multiple therapies before ^131^I-MIP-1095 including chemotherapy in many cases (Supplementary data Table B). The onset of myelosuppression occurred within 6 weeks post treatment with quite variable time to recovery, in some cases requiring up to 3–6 months for recovery. White blood cells typically recovered within several weeks, while platelets required several months to recover.

No significant changes in renal function were noted across patients after treatment (Fig. 5). Based on normal tissue tolerance limits for external beam radiotherapy as described by Emami et al. [23], these absorbed dose estimates suggest that administered activities of up to 15 GBq (428 mCi) can be given based on kidney absorbed dose limits of 23 Gy. However, this value was derived from external beam radiotherapy and does not predict renal toxicity associated with radiotherapeutics. Correction of these data for patient-specific organ volume, adjustment or the linear-quadratic dose rate effect and expression in terms of biologic effective dose (BED) finally delivered values correlating with renal toxicity. With these methods, a total limit not to exceed 37 Gy BED to the kidneys has been proposed (for a detailed discussion and data obtained, see Supplementary information and Supplementary Table F). The maximum administered activity given in a single administration to stay below 37 Gy BED to the kidneys in our patients would range from 298 mCi to 1092 mCi (11 to 40 GBq) with a mean value of 668 mCi (24.7 GBq) as listed in Table 2 of the Supplementary information. With regard to a future dose escalation study, it has to be stated that a 2 mCi/kg (LBM) starting dose for the first cohort is at the lower end of the range of administered activity in our patients (Table 1 of Supplementary information) and provides room for a reasonable escalation of the subsequent cohorts and/or multiple doses of ^131^I-MIP-1095. Furthermore, liver function and thyroid hormone values did not change after treatment.

The intense accumulation of ^131^I-MIP-1095 in the salivary and parotid glands suggest that these glands may likely be affected by therapy. Seven patients have reported dry mouth and one patient was reported to have mucositis. These symptoms were transient in nature and resolved 3 to 4 weeks after treatment. However, we cannot exclude that these symptoms will be accentuated or even may not recover when more cycles are given. We attempted to reduce the uptake of the radiopharmaceutical by the glands by use of cold packs and lemon juice. It is not clear what, if any, impact these prophylactic measures had on an individual patient’s glandular function, and further clinical investigation is needed. Since PSMA is expressed constitutively in the salivary glands, the tracer binds to this target and it is rather improbable that lemon juice will have an impact on the local absorbed dose. This may include attempts at displacement of the tracer from the salivary glands by administration of the unlabeled compound or competing alternative PSMA inhibitors after peak tumor uptake has been reached. Since, in tumors, the ligands are internalized after binding, this displacement strategy should not influence tumor accumulation or retention. However, it is not clear yet whether the PSMA ligands are handled differently in normal organs such as salivary glands or kidneys than in tumors.

Administration of therapeutic activities of ^131^I-MIP-1095 demonstrated high levels of tumor uptake and prolonged retention as noted in Fig. 3. This is a prerequisite for a successful therapeutic application. Therapeutic efficacy was demonstrated (1) by symptom relief and (2) by a decrease in PSA values: out of 13 patients with bone pain, three patients had no pain and eight had a decrease in pain severity after therapy. For two patients no information was available. With regard to the PSA values 61 % had a decline of 50 % or more whereas 75 % had a drop off 30 % or greater (Fig. 6). One patient showed a long lasting complete response (Fig. 7). These effects on PSA were not related to the activity given to the patients, indicating that several biological features are influencing therapeutic efficacy such as differences in repair mechanisms in different metastases and varying protecting factors excreted from bone marrow nearby of bone metastases. Since the patients in this evaluation were mostly presenting with a huge tumor burden, relapse of the disease was expected. The median time to PSA progression was 126 days, indicating that multiple therapy cycles may be necessary to obtain a long lasting response. However, this may be associated with more pronounced side effects, especially concerning the salivary glands. Bear in mind that most of these patients will have chemotherapy before we may expect that the pre-damaged bone marrow will be the dose-limiting organ in a considerable number of patients. This problem is shown by the data presented in Fig. 4: 17 of 21 patients had erythrocyte values below normal before therapy.

In general, we observed that lymph node metastases showed a better response than bone metastases, but the number of patients with lymph node metastases only was too small (only two patients, Figs. 7 and 8c, d) to draw a conclusion on that matter. Since bone metastases from prostate cancer are usually osteoblastic we may expect absorption of the beta particles leading to a reduced cross-fire effect in these lesions. Furthermore, as mentioned above, protective factors may be secreted by the surrounding bone or bone marrow.

## Conclusion

The systemic therapy with cytotoxic drugs causes side effects in the proliferating organs and it is anticipated that targeted drugs are free of these effects. However, targeted drugs may have the side effects seen in the excreting organs and in off-target organs with expression of the target. Moreover, when treating the enormous masses of tumor tissue found in the otherwise incurable patients, collective activities that threaten other organs have to be applied. Consequently, dosimetric studies are imperative prior to the application of novel targeted endoradiotherapeutic drugs.

The preliminary findings presented here of 28 patients with late-stage hormone- and chemotherapy-refractory prostate cancer after PSMA-targeted systemic radiotherapy are encouraging. In most cases there was extensive skeletal and soft tissue involvement of their cancer and most patients were clinically anemic at the time of treatment due to the stage of their disease and treatments they had endured.

To date, the results of both the ^124^I diagnostic and ^131^I therapeutic administration have shown that the treatment was well-tolerated, allowing for the estimation of absorbed dose to normal organs, and the patients demonstrated objective clinical measures of improvement in their condition. Preliminary efficacy was demonstrated by PSA reduction, a general observation of a reduction in bone pain and improved quality of life, and radiographic reductions in disease burden as evidenced by reduction in lesion size, extent and number of lesions as seen on the diagnostic scans. This has to be compared to the efficacy obtained with carbazitaxel [24], the current gold standard for the treatment of mCRPC. The limited efficacy of this compound, together with its strong side effects can be explained as it is known that taxanes, similar to other, non-targeted cytotoxic drugs do not show specific uptake [25].

Additional acute toxicities that were felt to be treatment-related included radiation-induced xerostomia and one case of hypothyroidism in a patient who experienced nausea and emesis after receiving the thyroid blocking agent prior to treatment. Because of the concern regarding the potential for radiation toxicity to the kidneys as a possible chronic long-term side effect of ^131^I-MIP-1095 therapy, available clinical data were collected that assessed renal function. While the data are inherently limited in terms of long-term follow-up, as the treatment of most of these patients provides less than a year of follow-up, there is no apparent evidence or negative trend in either calculated GFR or serum creatinine levels, which is consistent with the absorbed dose estimates to kidneys.

Overall, the patients tolerated the PSMA-targeted molecule treatments well. Myelosuppression was the primary and most significant side effect and was seen in most of the patients, varying in degree from mild to moderate. However, these patients had multiple treatments including chemotherapy before and many presented with values below normal already at baseline.

It has to be emphasized that, in our view, most patients preferred this form of treatment over chemotherapy to help control their disease. Most patients have reported a reduction in bone pain and an improved quality of life and mental state since receiving the PSMA-targeted molecule therapy. These encouraging preliminary results warrant the formal evaluation of the ^131^I-labeled small molecule inhibitor of PSMA in controlled clinical studies.

## Electronic supplementary material

Below is the link to the electronic supplementary material.

ESM 1 (DOCX 33 kb)

ESM 2 (DOCX 17 kb)

ESM 3 (DOC 81 kb)

ESM 4 (DOC 29 kb)

ESM 5 (DOC 20 kb)

ESM 6 (DOC 24 kb)

ESM 7 (PDF 41 kb)
