# Supplementary material for: Radiation dosimetry and first therapy results with a 124I/131I-labeled small molecule (MIP-1095) targeting PSMA for prostate cancer therapy
Source: Eur J Nucl Med Mol Imaging. 2014 Feb 28;41(7):1280–92. doi: 10.1007/s00259-014-2713-y (PMC4052014; doi:10.1007/s00259-014-2713-y)
Supplement: Supplementary file 1 — (DOCX 33 kb) [file 259_2014_2713_MOESM1_ESM.docx]

Emami EBRT-Derived Limits and Medical Internal Radiation Dose (MIRD) Schema

A comprehensive review of follow-up reports of patients receiving external beam radiation therapy (EBRT) published by Emami et al observed that the probability of toxicity within a five year period^1^ may be predicted when the kidneys receive radiation absorbed doses greater than 23 Gy. This limit therefore is commonly utilized in clinical practice with EBRT and with systemically delivered unsealed source radiotherapies. Unfortunately, this well-established limit for EBRT has not consistently predicted renal toxicities, or lack thereof, associated with the low molecular-weight, systemically delivered radiotherapeutics. In previous clinical experience with ^90^Y-DOTATOC^2,3^ and ^166^Ho-DOTMP^11^, the standard Medical Internal Radiation Dose (MIRD) schema^12^ for calculating radiation dosimetry did not correlate with clinical manifestations of renal disease. Therefore, a more accurate and reliable image-based renal dosimetry model was needed for targeted radiotherapeutic applications in order to more accurately assess any dose-effect relationship with toxicity and serve as a tool for prediction of dose related toxicity.

In response to the growing need for improved renal dosimetry, the MIRD committee published several enhancements to the standard kidney model. The most significant of these was to allow for the substitution of measured, patient-specific kidney volumes^13^ for the standard kidney volume described in the original MIRD schema^12^. Subsequent refinements included replacing the simple ellipsoid geometry with several different sub-organ structures of the kidney^14^ and the addition of Linear-Quadratic (L-Q) techniques^15^. The latter allow for modeling of the protracted and exponentially decaying dose rate and its linear relationship to repairable single-strand DNA breaks and non-linear association with irreparable double-strand breaks.

Biologic Effective Dose

Output of the improved MIRD kidney model could now be translated into Biologic Effective Dose (BED). This unit of radiation exposure is commonly used in EBRT to compare different radiation absorbed doses across different rates and fractioning schemes. When put in terms of BED, exposures from targeted radiotherapies can be compared with those from EBRT.

Considerable work has been published describing the dose-effect relationship with ^90^Y-DOTATOC targeted radiotherapy and renal toxicity^2-10^. As expected, absorbed dose estimates to the kidneys derived by using conventional dosimetry techniques did not correlate with clinical renal impairment. However, as published in the Journal of Nuclear Medicine (Barone et al 2005), when these data were corrected for patient-specific organ volume, adjusted for the linear-quadratic dose-rate effect and expressed in terms of BED, a strong and significant correlation was observed^2,3^. Moreover, the BED values and observations of renal toxicity appear to also show agreement with EBRT organ tolerance values when put in terms of BED. These analyses suggest an upper limit of 37 Gy BED to the kidneys.

Application of Biologic Effective Dose (BED) Analysis to Previous Experience with ^131^I-MIP-1095

Our data collected in the physician-directed clinical treatment program suggest a 37 Gy total BED exposure limit and a starting dose of 2 mCi/kg of lean body mass (LBM) are both appropriate and safe. BED dose estimates for ^131^I based on PET/CT images of the ^124^I PSMA-targeted compound used in Heidelberg are listed in Table 1. In patients with pre-therapy ^124^I PET/CT dosimetry followed by ^131^I therapy, initial treatments ranged from 92 to 194 mCi ( 3.4 to 7.1 GBq). The maximum administered activity given in a single administration to stay below 37 Gy BED to the kidneys in our patients would range from 298 mCi to 1092 mCi ( 11 to 40 GBq) with a mean value of 668 mCi (24.7 GBq) as listed in Table 2. A 2 mCi/kg (LBM) starting dose for the first cohort is at the lower end of the range of administered activity in our patients (Table 1) and provides room for a reasonable escalation of the subsequent cohorts and/or multiple doses of ^131^I-MIP-1095.

Table 1. Patients with ^124^I PET/CT Dosimetry and Treatment with I-131 Labeled PSMA-targeting Compound (1^st^ cycle).

| **Patient** | **LBM** | **Admin** | **Admin/LBM** | **Dose (phantom)** | **Dose (actual)** | **BED** |
| --- | --- | --- | --- | --- | --- | --- |
| **ID** | **(kg)** | **(mCi)** | **(mCi/kg)** | **(Gy)** | **(Gy)** | **(Gy)** |
| 07 | 59.6 | 194 | 3.3 | 2.9 | 2.8 | 4.7 |
| 08 | 60.5 | 184 | 3.0 | 17.1 | 7.5 | 11.3 |
| 13 | 59.6 | 170 | 2.9 | 11.4 | 4.5 | 7.0 |
| 04 | 57.7 | 162 | 2.8 | 13.9 | 9.5 | 17.1 |
| 09 | 58.2 | 146 | 2.5 | 10.7 | 6.0 | 9.1 |
| 03 | 72.7 | 181 | 2.5 | 7.7 | 3.5 | 5.5 |
| 06 | 65.0 | 159 | 2.5 | 11.0 | 7.2 | 10.9 |
| 01 | 63.0 | 135 | 2.1 | 4.6 | 3.4 | 5.4 |
| 11 | 76.0 | 162 | 2.1 | 12.0 | 6.9 | 11.3 |
| 15 | 67.3 | 143 | 2.1 | 7.2 | 3.1 | 4.8 |
| 16 | 82.0 | 162 | 2.0 | 10.0 | 5.0 | 8.3 |
| 10 | 75.0 | 127 | 1.7 | 4.2 | 3.3 | 5.2 |
| 05 | 59.3 | 95 | 1.6 | 1.0 | 1.1 | 1.7 |
| 02 | 66.4 | 92 | 1.4 | 2.0 | 2.0 | 3.0 |
| *Min* | *57.7* | *92* | *1.4* | *1.0* | *1.1* | *1.7* |
| *Max* | *82.0* | *194* | *3.3* | *17.1* | *9.5* | *17.1* |
| *Median* | *64.0* | *161* | *2.3* | *8.9* | *4.0* | *6.3* |

LBM= Lean Body Mass; Admin= Administered Dose; Dose(phantom)=Kidney radiation dose from OLINDA for total administered activity based on phantom-based kidney mass; Dose(actual)=Kidney radiation dose adjusted for patient-specific kidney mass; BED=Biologic Effective Dose.

Table 2. Estimated Activity of a Single Administration of ^131^I labeled PSMA-targeted Compound Required to Achieve 37 Gy BED to the Kidneys.

| **Patient ID** | **Admin**  **(mCi)** |
| --- | --- |
| 07 | 933 |
| 04 | 298 |
| 05 | 1092 |
| 02 | 806 |
| 01 | 691 |
| 03 | 865 |
| 10 | 658 |
| 09 | 513 |
| 11 | 435 |
| 06 | 482 |
| 16 | 544 |
| 08 | 540 |
| 15 | 789 |
| 13 | 711 |
| *Mean* | *668* |
| *SD* | *216* |

**References**

1. Emami B, Lyman J, Brown A, et al. Tolerance of normal tissue to therapeutic irradiation, *Int J Radiat Oncol Biol Phys* 1991; 21:109-122.
2. Barone R, Borson-Chazot F, Valkema R, et al. Patient-specific dosimetry in predicting renal toxicity with ^90^Y-DOTATOC: relevance of kidney volume and dose-rate in finding a dose–effect relationship. *J Nucl Med.* 2005;46(suppl):99S–106S.
3. Bodei L, Cremonesi M, Ferrari M, et al. Long-term evaluation of renal toxicity after peptide receptor radionuclide therapy with ^90^Y-DOTATOC and ^177^Lu-DOTATATE: the role of associated risk factors. *Eur J Nucl Med Mol Imaging*. 2008;35:1847–1856.
4. Van Binnebeek S, Baete K, Terwinghe C, Vanbilloen B, Haustermans K, Mortelmans L, Borbath I, Van Cutsem E, Verslype C, Mottaghy FM, Verbruggen A, Deroose CM. [Significant impact of transient deterioration of renal function on dosimetry in PRRT.](http://www.ncbi.nlm.nih.gov/pubmed/22961123) *Ann Nucl Med.* 2012; Sep 9. [Epub ahead of print]
5. Valkema R, Pauwels SA, Kvols LK, Kwekkeboom DJ, Jamar F, de Jong M, Barone R, Walrand S, Kooij PP, Bakker WH, Lasher J, Krenning EP. [Long-term follow-up of renal function after peptide receptor radiation therapy with (90)Y-DOTA(0),Tyr(3)-octreotide and (177)Lu-DOTA(0), Tyr(3)-octreotate.](http://www.ncbi.nlm.nih.gov/pubmed/15653656) *J Nucl Med.* 2005 Jan;46 Suppl 1:83S-91S.
6. Stahl A, Schachoff S, Beer A, Winter A, Wester HJ, Scheidhauer K, Schwaiger M, Wolf I. [[(111)In]DOTATOC as a dosimetric substitute for kidney dosimetry during [(90)Y]DOTATOC therapy: results and evaluation of a combined gamma camera/probe approach.](http://www.ncbi.nlm.nih.gov/pubmed/16645839) *Eur J Nucl Med Mol Imaging*. 2006;33(11):1328-36.
7. Konijnenberg M. Is the renal dosimetry for (90)Y-DOTA(0),Tyr(3)-octreotide accurate enough to predict thresholds for individual patients? *Cancer Biother. Radiopharm*. 2003;18:619–625.
8. Lambert B, Cybulla M, Weiner S, Van De Wiele C, Ham H, Dierckx R, Otte A. Renal toxicity after radionuclide therapy. *Radiat. Res.* 2004;161:607–611.
9. Konijnenberg M, Melis M, Valkema R, Krenning E, de Jong M. Radiation dose distribution in human kidneys by octreotides in peptide receptor radionuclide therapy. *J. Nucl. Med*. 2007;48:134–142.
10. Sgouros G. Dosimetry of Internal Emitters. *J Nucl Med*. 2005;46:18S–27S.
11. Breitz H, Wendt R, Stabin M, Bouchet L, Wessels B. Dosimetry of high dose skeletal targeted radiotherapy (STR) with ^166^Ho-DOTMP. *Cancer Biother Radiopharm.* 2003;18:225–230.
12. Snyder WS, Ford MR, Warner GG. *Estimates of Specific Absorbed Fractions for Photon Sources Uniformly Distributed in Various Organs of a Heterogeneous Phantom. MIRD Pamphlet No. 5, revised*. New York, NY: Society of Nuclear Medicine; 1978.
13. Siegel J, Thomas S, Stubbs J, Stabin M, et al. MIRD Pamphlet No. 16: Techniques for Quantitative Radiopharmaceutical Biodistribution Data Acquisition and Analysis for Use in Human Radiation Dose Estimates. *J Nucl Med* 1999; 40:37S-61S.
14. Bouchet LG, Bolch WE, Blanco HP, et al. MIRD pamphlet No. 19: Absorbed fractions and radionuclide S values for six age-dependent multiregion models of the kidney. *J Nucl Med* 2003;44:1113.
15. Wessels B, Konijnenberg M, Dale R, Breitz H, Cremonesi M, Meredith R, Green A, Bouchet L, Brill B, Bolch W, Sgouros G, Thomas S. MIRD Pamphlet No. 20: MIRD Pamphlet No. 20: The Effect of Model Assumptions on Kidney Dosimetry and Response – Implications for Radionuclide Therapy. *J Nucl Med* 2008; 49:1884-1899.
16. Baechler S, Hobbs RF, Prideaux AR, Wahl RL, Sgouros G. Extension of the biological effective dose to the MIRD schema and possible implications in radionuclide therapy dosimetry. *Med Phys.* 2008;35:1123–1134.
17. Bodey R, Evans P, Flux G. Application of the linear-quadratic model to combined modality radiotherapy. *Int. J. Radiat. Oncol. Biol. Phys.* 2004;59: 228–241.
18. Stabin M, Sparks R, Crowe E. OLINDA/EXM: The Second-Generation Personal Computer Software for Internal Dose Assessment in Nuclear Medicine. *J Nucl Med* 2005; 46:1023-1027.
19. [Baechler S](http://www.ncbi.nlm.nih.gov/pubmed?term=Baechler%20S%5BAuthor%5D&cauthor=true&cauthor_uid=23039651), [Hobbs RF](http://www.ncbi.nlm.nih.gov/pubmed?term=Hobbs%20RF%5BAuthor%5D&cauthor=true&cauthor_uid=23039651), [Boubaker A](http://www.ncbi.nlm.nih.gov/pubmed?term=Boubaker%20A%5BAuthor%5D&cauthor=true&cauthor_uid=23039651), [Buchegger F](http://www.ncbi.nlm.nih.gov/pubmed?term=Buchegger%20F%5BAuthor%5D&cauthor=true&cauthor_uid=23039651), [He B](http://www.ncbi.nlm.nih.gov/pubmed?term=He%20B%5BAuthor%5D&cauthor=true&cauthor_uid=23039651), [Frey EC](http://www.ncbi.nlm.nih.gov/pubmed?term=Frey%20EC%5BAuthor%5D&cauthor=true&cauthor_uid=23039651), [Sgouros G](http://www.ncbi.nlm.nih.gov/pubmed?term=Sgouros%20G%5BAuthor%5D&cauthor=true&cauthor_uid=23039651). Three-dimensional radiobiological dosimetry of kidneys for treatment planning in peptide receptor radionuclide therapy. [*Med Phys*.](http://www.ncbi.nlm.nih.gov/pubmed/23039651) 2012;39(10):6118-6128.
