# Supplementary material for: Radiation dosimetry and first therapy results with a 124I/131I-labeled small molecule (MIP-1095) targeting PSMA for prostate cancer therapy
Source: Eur J Nucl Med Mol Imaging. 2014 Feb 28;41(7):1280–92. doi: 10.1007/s00259-014-2713-y (PMC4052014; doi:10.1007/s00259-014-2713-y)
Supplement: Supplementary file 2 — (DOCX 17 kb) [file 259_2014_2713_MOESM2_ESM.docx]

**Supplementary data table A: patient characteristics**

| **Pat.No** | **I124  [in MBq]** | **age  [in year]** | **PSA [in ng/ml]** | **No. of scans** | **T1 [in h]** | **T2 [in h]** | **T3 [in h]** | **T4 [in h]** | **T5 [in h]** |
| --- | --- | --- | --- | --- | --- | --- | --- | --- | --- |
| 1 | 56.0 | 73.2 | 126.00 | 5 | 1.18 | 3.43 | 19.45 | 72.05 | 92.48 |
| 2 | 26.0 | 64.4 | 209.00 | 5 | 0.51 | 2.97 | 17.38 | 41.23 | 65.13 |
| 3 | 48.2 | 56.3 | 50.70 | 5 | 1.03 | 4.05 | 18.38 | 42.38 | 68.38 |
| 4 | 45.0 | 71.3 | 201.00 | 4 | 1.00 | 4.72 | 19.68 | 42.65 | n.a. |
| 5 | 55.7 | 73.3 | 339.00 | 3 | 1.51 | 17.76 | 42.40 | n.a. | n.a. |
| 6 | 52.8 | 63.4 | 577.00 | 5 | 1.08 | 5.35 | 20.17 | 44.00 | 73.02 |
| 7 | 66.3 | 70.2 | 411.00 | 5 | 1.05 | 4.17 | 18.30 | 42.77 | 69.30 |
| 8 | 69.2 | 71.1 | 186.00 | 5 | 1.18 | 3.96 | 19.58 | 43.33 | 67.52 |
| 9 | 59.6 | 75.6 | 114.00 | 5 | 1.73 | 4.33 | 19.67 | 43.75 | 67.67 |
| 10 | 77.5 | 68.2 | 1.13 | 5 | 0.98 | 3.58 | 20.22 | 44.65 | 68.18 |
| 11 | 68.0 | 76.2 | 27.20 | 5 | 0.85 | 2.43 | 18.30 | 42.55 | 66.58 |
| 12 | 105.0 | 73.5 | 98.90 | 5 | 1.80 | 4.28 | 18.08 | 41.60 | 67.72 |
| 13 | 98.0 | 76.6 | 107.00 | 5 | 1.32 | 3.98 | 20.15 | 44.20 | 116.32 |
| 14 | 94.0 | 55.2 | 31.40 | 5 | 1.32 | 4.00 | 18.80 | 42.85 | 114.82 |
| 15 | 75.0 | 74.8 | 4.58 | 5 | 1.35 | 4.48 | 18.17 | 42.10 | 66.15 |
| 16 | 82.2 | 72.5 | 14.30 | 3 | 1.12 | 50.00 | 68.15 | n.a. | n.a. |
|  |  |  |  |  |  |  |  |  |  |
| median | 67.15 | 71.90 | 110.50 | 5 | 1.15 | 4.10 | 19.51 | 42.80 | 68.18 |
| min | 26.00 | 55.20 | 1.13 | 3 | 0.52 | 2.43 | 17.38 | 41.23 | 65.13 |
| max | 105.00 | 76.58 | 577.00 | 5 | 1.80 | 50 | 68.15 | 72.05 | 116.32 |
