# Supplementary material for: Radiation dosimetry and first therapy results with a 124I/131I-labeled small molecule (MIP-1095) targeting PSMA for prostate cancer therapy
Source: Eur J Nucl Med Mol Imaging. 2014 Feb 28;41(7):1280–92. doi: 10.1007/s00259-014-2713-y (PMC4052014; doi:10.1007/s00259-014-2713-y)
Supplement: Supplementary file 3 — (DOC 81 kb) [file 259_2014_2713_MOESM3_ESM.doc]

| Patient # | Date of PCa Dx | Type and Date of Initial Tx | Secondary Tx and Dates | Hormone Tx and Dates | Chemo Tx and Dates | PSMA Tx Dates |
| --- | --- | --- | --- | --- | --- | --- |
| PSMA01 | 2001 pT2a pN1 (1/15) pMx G3a G 7 (4+3) | RPr + LAD 2001 | RT Th 9 (10 Gy) 9-10/10) | Casodex 10/04  Trenantone 8/08  Trenantone + Bicalutamid 10/10  Estraderm TTS (off label) 4/11 | Chemo naïve | 5 GBq 7/1/11 |
| PSMA02 | Dec 04  pT3b N1 L1 R1  G 8 (4+4) | RPr + LAD  July 20, 05 | EXBT prostate (65 Gy) and pelvic nodes (45 Gy) 11/05  Sternum 1/07 (30 Gy)  6th right rib + 9th left rib 2/07 (30 Gy)  C Spine 7/08  rt trochanter 12/08 (30 Gy)  T Spine (3rd + 7th) 2/10 (20.6 Gy)  Rt shoulder 5/10 (8 Gy) | Casodex 12/04  Trenantone  Zometa  Arbiraterone 2/12 | Docetaxel 8/05  Mitoxantron 2x 11/05  Docetaxel 1/06  Docetaxel 6/06  Vaccination Tx 4/09  CTLA4 (Ipilimumab vs. placebo) 5-12/10  Docetaxel 3x 12/10  Docetaxel 3x 2-3/11 | 3.4 GBq Aug 2, 11 |
| PSMA03 | Oct 06  cT3a cN0 M0,  G 9 (4+5)  PSA 5,5 | High Dose rate brachytherapy 1/07 and 2/07 | Salavage RPr and node dissection 10/09  pT3b pN1 (29/37) pMx L1  Bladder invasion 4/11  Bladder resection 4/11 | Bicalutamid 08  LHRH (Casodex) 10/09  Trenantone 4/10  Bicalutamid 4/10  Duastosterid 4/10 | Docetaxel 11/10  Docetaxel 3/11 | 6.7 GBq Sept 13, 11 |
| PSMA04 | July 06  TNM n.a. (only biopsy)  G 6 (3+3)  PSA 20 | 72 Gy EXBRT 06, no drop in PSA | None | LHRH, BS Bicalutamide  Antiandrogen therapy from 06-10. Eventually became castrate resistant | Chemo naïve | 6 GBq Sept 28, 11 |
| PSMA05 | 1/97  Originally T2, revised T3  N0  G 7 (3+4) | RPr 1/97  lymph node dissection 99 | EBRT 11/99 | ADT for 5 years  Abiraterone | Docetaxel 11/04  Docetaxel 4/05  Docetaxel 3/07  Docetaxel 6/07  Docetaxel 8/09  Docetaxel 12/09  Xeloda and Sutent 9/08  Xeloda and Sutent 2/09 | 3.5 GBq Sept 29, 11 |
| PSMA06 | 09/01/06  pT2c, N0, Mx, R1  G 8 (5+3) | RPr 11/06 | No RT due to lymph nodes met diagnosis 06/2008 | Flutamid 06/08  Trenantone 04/09 both stopped due to side effects,  Abiraterone with possible hydropic decompensation of kidney | 6x Docetaxel since 09/10 to 11/10 | 5.9 G GBq Nov 2,11 |
| PSMA07 | PSA in 6/05  Up to 23.5 – no biopsy.  diagnosis of bone met in 11/07 | Initially viscum therapy and anthroposophic therapy  No RT or RPr | ADT | 04/08 PSA 1329 begin Zoladex  Casodex 3-4/08  Avodart 09/09  Estradurin 11/10-3/11  Bicalutamid 6/11-7/11  Pamorelin 7/11 | Chemo naïve | 7.2 GBq Aug 11, 11 |
| PSMA08 | 11/01  pT3a pNx M0 G2,  PSA 5.8, G 6 | RPr 11/01 | EBRT 5-7/06 (66 Gy) | Several ADT  Avodart  Abiraterone 9/11 | 6x Chemo 7/12/10 | 6.8 GBq Nov 22, 11 |
| PSMA09 | 12/05 T1c N0 M0  G 7(3+4) PSA 25.7 | ADT, no RPr | IMRT 06-09/06 (76 Gy)  IMRT + IGRT pelvic lymph nodes (468 Gy) with integrated boost on left sided LN (57.2 Gy) 07-09/2010 | Trenantone until 06/06 with single doses in 07 + 09 again 2/10 | Chemo naïve | 5.4 GBq Dec 20, 11 |
| PSMA10 | 2009  pT3b, pN1 (9/45), G3, R1  G 9 PSA 16.9 | RPr + LAD 3/10 | EBRT 8/10 Pelvis + LAD (66 Gy) | ADT med before PSMA | Chemo naïve | 4.7 GBq Jan 16, 12 |
| PSMA11 | 2/1999  pT3a pN1 M0 G3 R0  G 9  PSA 29,5 | RPr 99 | EBRT 2-4/98 Pelvis (45 Gy) + L5 (39 Gy)  EBRT 4-5/09 L1-4 (40 Gy) | Leuprorelin 4/00-3/01  Leuprorelin + Bicalutamid 12/02-11/03 + 5-11/05  Bicalutamid 1-3/09  Bicalutamid 11/09-12/10  8/10 Goserelin  Abiraterone 12/11- | 4x Docetaxel 04-06/11  (stopped due to polyneuropathia) | 3.5 GBq Feb 7, 12 |
| PSMA12 | 3/03 | RPr 3/03 | EBRT 6-7/03 (64,8 Gy)  tomotherapy sacrum 7-8/07 (60 Gy)  Right pelvis 1/10 (30 Gy)  tomotherapy acetabulum + SIAS right 11/10 (37 Gy)  IMRT TH 8, 7^th^ rib right, L3, Os ilium right (36 Gy)  RT with C12 acetabulum right (36 Gy) | Induction phase Ipilimumab vs. Placebo 5/11-10/11 + LHRH  Trenantone | Chemo naïve | 5.35 GBq Feb 23, 12 |
| PSMA13 | 09/04  pT3b N1 (4/16), R1 V1 L1,  G 7 (4+3)  PSA 5.7 | RPr 09/04 | EBRT pelvis and bone 03/08 (63 Gy) | Casodex 12/07 – 09 or 2010  Zoladex  MDV3100 since 7/10 | 6x Docetaxel 09/09-02/10 | 6.3 GBq Feb 28,12 |
| PSMA14 | 2/06  TNM n.a.  G 5 + PIN  PSA 12.6 | Brachytherapy 6/06 | EBRT 9-10/10 (35 Gy) | Zoladex + Casodex 12/09 – 09/10  Trenantone + initial 14d Bicalutamid 2/12 | Chemo naïve | 6.0 GBq Feb 29, 12 |
| PSMA15 | 4/01  pT2a, pN0, G2  G 7 (3+4) | RPr 4/3/01 | EBRT 07 (63 Gy reduced due to PSA progress at 45 Gy – from 0.52 to 0.71 ng/dl) | Zoladex + Casodex 08  Trenantone + Casodex 09 + again 10-11  MDV3100 (placebo?) |  | 5.3 GBq Mar 6, 12 |
| PSMA16 | 10/11  pT3b pN1 (6/11) R2 (extrapelvine LN)  G 9(4+5)  PSA 104 | PRr non-nerve-sparing + LAD 11/21/11 | No RT since extrapelvic LN  Therefore AHT | Bicalutamid 9/11  Trenantone 9/12 | Chemo naïve | 6.0 GBq Mar 19,12 |
| PSMA17 | 6/11  T4, N2, M2  G 8-9  PSA 5.5 | ADT | EBRT prostate 7/27/11  EBRT T1 01-02/12 | Casodex + Firmagon 6/11  Denosumab | Docetaxel | 3.7 GBq Apr 4, 12 |
| PSMA18 | 4/04  pT3b pN0 cM0 L1  G 7 (3+4)  PSA 9.4 | RPr + LAD 4/04  Re-OP with rectum, bladder + LAD 2/17/12 | EBRT prostate (66Gy) 3/06 | Trenantone 2008-09  + Casodex 2009-11 | (6x R-CHOP due to B-NHL) | 3.7 GBq May 4, 12 |
| PSMA19 | 3/07  pT2c pN1  G 7 (4+3)  PSA 22 | RPr + bladder 6/07 | EBRT left pelvis 3/08 (40 Gy)  Re-RT left pelvis 3/09 (30 Gy) | Primary and secondary AHT incl. Ketoconazol  Abiraterone | 19x Docetaxel  Carboplatin  TKI (Sunitinib and Sorafenib)  Cabazitaxel | 4 GBq May 7, 12 |
| PSMA20 | 2001  pT2b G2  G 5 (2+3) second look G 7 (4+3)  PSA 20 | EBRT pelvis + seminal vesicle 2-4/09 (76 Gy) – stop AHT | Hyperthermia and cell symbiosis (holistic in 04 + 08)  Enzymatic therapy | Prostasol, then Trenantone 01  Flutamid + Trenantone + Prostasol 8/08  Androcur  Abiraterone | Chemo naive | 6 GBq May 15, 12 |
| PSMA21 | 9/09  G 9 (5+4)  PSA 4.9 | ADT | No OP, no RT | Trenantone, then  Bicalutamid 3/12- | Chemo naïve | 4.8 GBq June 6, 12 |
| PSMA22 | 8/1998  Biopsy G 7,  pT3a Nx cM0 R1  G 8 (5+3)  PSA 11.9 | RPr 9/1998 | AHT | - Intermittent AHT 98-00  - Triple AHT 00-02  - triple AHT 6/03  - 12/06 modif. triple AHT  - 11/10 modif. triple AHT  - 4-7/11 with Abiraterone PD | - metronomic low dose chemo 11/03-4/04  - triple AHT 11/03-11/05  - thalidomide 11/03-3/08  - mod. triple AHT 12/06-10/07  - ketoconazole 1/08-2/09  - ketoconazole 6/09-3/10  - ketoconazole 12/11-  - 7/10 experimental Th with dendritic cells (LANEX)  - Zoladex + Proscar or Avodart 1/08-2011 | 2 GBq June 21, 12 |
| PSMA23 | 8/2007  pT3b N1 M1 R0  G 9  PSA 345 | RPr | RT of left hip (for stability and pain reasons) 4/09 | Leuprorelin since 8/07  Bicalutamid 5-7/08 | 6+6+4x Docetaxel 7/08-2/10  Second line chemo Mitoxantron/prednisolone 2/10-6/10  AFFIRM study (MDV 3100) in placebo arm  Third line chemo with 6x Cabacitaxel 3/11-6/11  Abiraterone since 9/11-2/12  4x Cabacitaxel 3-5/12  Since June 16, 12 MDV3100 | 3 GBq June 28, 12 |
| PSMA24 | 3/2009  pN1 (1/5)  PSA 50 | RPr 3/09 | RT left pelvis | 7/09 Trenantone  10/09 Casodex | 10x Docetaxel 9/10 – 3/11 | 2,5 GBq Jul 10, 12 |
| PSMA25 | 6/2005  pT3 pN1 (1/25) M0 R1  G 7 (3+4)  PSA: 329 | RPr 6/05 | EBRT pelvis (64 Gy) 9-11/05 | - 2006-08 Trenantone  - 10/08-03/09 total androgen block  - 3/09-10/09 LhRh  - 10/09-04/10 total androgen block  - from 4/10 Androcur | Chemotherapy | 2 GBq Jul 17, 12 |
| PSMA26 | 1/2011  G 10 | RPr 8/11 | EBRT 8/11 | Trenantone since 1/11 | 4x Docetaxel + Carboplatin (until 8/12) | 3.7 GBq Sep 18, 12 |
| PSMA27 | 6/2000  primarily metastasized prostate cancer (bone, lymph nodes) PSA 270 | No OP | No RT | Primary AHT, currently Zoladex and since 3/11 Abiraterone | Chemo naïve | 3 GBq Sep 25, 12 |
| PSMA28 | 11/2001  G 7  PSA 569 | No OP | - RT of acetabulum and LWS  - Samarium with 3.4 GBq 5/11 | Primary AHT (Trenantone, Bicalutamid)  - since 1/12 Abiraterone (intermittent) | 2x Docetaxel 3/10 – 10/10 (stopped due to PD) | 2.3 GBq Nov 11, 12 |
