# Supplementary material for: Radiation dosimetry and first therapy results with a 124I/131I-labeled small molecule (MIP-1095) targeting PSMA for prostate cancer therapy
Source: Eur J Nucl Med Mol Imaging. 2014 Feb 28;41(7):1280–92. doi: 10.1007/s00259-014-2713-y (PMC4052014; doi:10.1007/s00259-014-2713-y)
Supplement: Supplementary file 4 — (DOC 29 kb) [file 259_2014_2713_MOESM4_ESM.doc]

**Supplementary data table C: SUV in tumor lesions and normal organs**

SUVmean in 115 tumor lesions

1h 4h 24h 48h 72h

Mean 6.95 8.79 11.14 11.05 8.5630

Median 5.80 7.30 9.50 9.50 7.50

STD 3.75 4.92 6.81 8.29 4.50

SER 0.34 0.45 0.63 0.80 0.50

Min 1.40 2.50 3.00 3.00 2.30

Max 20.60 34.60 47.70 70.20 25.40

SUVmean in the lacrimal glands in 16 patients

1h 4h 24h 48h 72h

Mean 3.48 3.96 5.94 5.42 4.59

Median 3.20 3.90 5.00 4.70 3.90

STD 1.25 1.26 2.39 2.57 1.93

SER 0.32 0.32 0.61 0.66 0.58

Min 1.40 1.80 3.30 2.80 2.60

Max 5.70 6.70 10.30 11.10 8.50

SUVmean in the parotid glands in 16 patients

1h 4h 24h 48h 72h

Mean 7.71 9.87 11.50 9.94 7.62

Median 6.60 9.20 11.60 9.80 6.70

STD 2.92 3.67 3.93 3.99 2.57

SER 0.75 0.94 1.01 1.03 0.77

Min 2.50 2.60 2.85 1.70 5.40

Max 12.60 16.60 19.80 17.00 14.00

SUVmean in the submandibular glands in 16 patients

1h 4h 24h 48h 72h

Mean 7.70 10.26 12.01 9.72 6.53

Median 7.60 9.50 11.60 9.00 6.30

STD 2.06 3.549 4.78 4.72 2.47

SER 0.53 0.91 1.23 1.22 0.74

Min 4.60 4.60 5.50 4.10 3.10

Max 12.10 18.10 22.20 19.80 11.70

SUVmean in the liver in 16 patients

1h 4h 24h 48h 72h

Mean 5.36 5.95 5.89 4.20 3.13

Median 5.15 5.70 5.30 4.00 3.10

STD 1.53 1.83 2.29 1.28 0.58

SER 0.38 0.47 0.59 0.33 0.17

Min 3.60 3.20 2.90 1.60 1.90

Max 8.60 9.30 9.30 6.40 4.30

SUVmean in the spleen in 16 patients

1h 4h 24h 48h 72h

Mean 4.18 3.44 2.68 2.35 2.10

Median 3.85 3.30 2.90 2.50 2.60

STD 1.36 0.54 0.44 0.57 0.78

SER 0.34 0.13 0.11 0.14 0.23

Min 2.80 2.80 1.70 1.00 1.10

Max 7.90 5.00 3.30 3.10 3.00

SUVmean in the kidneys in 16 patients

1h 4h 24h 48h 72h

Mean 4.24 4.82 4.05 3.19 2.84

Median 4.10 4.50 4.00 3.20 3.00

STD 0.80 1.16 1.27 0.90 0.70

SER 0.20 0.29 0.32 0.23 0.21

Min 3.00 2.90 1.70 1.10 1.00

Max 5.40 6.90 6.20 4.60 3.90

SUVmean in gluteal muscle in 16 patients

1h 4h 24h 48h 72h

Mean 0.79 0.56 0.48 0.38 0.31

Median 0.80 0.60 0.40 0.40 0.20

STD 0.50 0.22 0.20 0.16 0.20

SER 0.12 0.05 0.05 0.04 0.06

Min 0.20 0.20 0.30 0.10 0.10

Max 2.40 1.00 1.00 0.60 0.70

SUVmean in the mediastinal blood pool in 16 patients

1h 4h 24h 48h 72h

Mean 1.51 1.29 0.81 0.71 0.57

Median 1.40 1.20 0.70 0.90 0.40

STD 0.75 0.59 0.55 0.36 0.36

SER 0.18 0.15 0.14 0.09 0.11

Min 0.60 0.60 0.01 0.01 0.20

Max 3.30 2.70 2.30 1.20 1.20
