# Supplementary material for: Radiation dosimetry and first therapy results with a 124I/131I-labeled small molecule (MIP-1095) targeting PSMA for prostate cancer therapy
Source: Eur J Nucl Med Mol Imaging. 2014 Feb 28;41(7):1280–92. doi: 10.1007/s00259-014-2713-y (PMC4052014; doi:10.1007/s00259-014-2713-y)
Supplement: Supplementary file 5 — (DOC 20 kb) [file 259_2014_2713_MOESM5_ESM.doc]

**Supplementary Table D: ratio of dosimetric values for ^131^I-MIP-1095 and values calculated from data published for ^90^Y-J591 and ^177^Lu-J591 [10,11].**

**^131^I-MIP-1095/ ^131^I-MIP-1095/**

**^90^Y-J591 ^177^Lu-J591**

adrenals 3.48

colon 11.63

small intestine 3.70

heart 0.19 0.61

kidneys 0.32 1.03

liver 0.22 0.7

lungs 0.13 0.52

red marrow 0.34 1.00

spleen 0.14 0.35

bladder 0.72 1.96

testes 0.99 2.98

effective dose 0.48 1.51
