# Supplementary material for: Radiation dosimetry and first therapy results with a 124I/131I-labeled small molecule (MIP-1095) targeting PSMA for prostate cancer therapy
Source: Eur J Nucl Med Mol Imaging. 2014 Feb 28;41(7):1280–92. doi: 10.1007/s00259-014-2713-y (PMC4052014; doi:10.1007/s00259-014-2713-y)
Supplement: Supplementary file 6 — (DOC 24 kb) [file 259_2014_2713_MOESM6_ESM.doc]

**Supplementary table E: Hematology prior (p) and after (a) therapy**

Pat no. GBq GBq/m^2^ WBCp WBCa Eryp Erya Platp Plata days

PSMA01 5.00 2.55 6.90 3.34 4.70 4.10 273 126 46

PSMA02 3.40 1.62 4.91 4.06 3.60 3.40 275 211 47

PSMA03 6.70 2.92 9.13 7.00 4.40 4.70 311 287 44

PSMA04 6.00 3.18 2.82 1.00 3.30 3.20 156 73 41

PSMA05 3.50 1.82 8.88 3.26 3.20 3.60 703 238 39

PSMA07 7.20 3.74 7.06 2.20 3.90 4.08 229 42 36

PSMA08 6.80 3.51 7.69 2.17 4.40 3.90 250 41 50

PSMA09 5.40 2.89 6.02 3.03 3.80 4.10 191 112 62

PSMA10 4.70 2.43 4.83 3.27 4.10 3.90 268 212 53

PSMA11 6.00 2.99 4.77 3.67 3.60 3.40 173 148 71

PSMA12 5.35 2.41 4.70 5.16 3.70 4.00 221 220 46

PSMA13 6.30 3.26 3.87 3.73 3.30 2.90 291 267 125

PSMA14 6.00 3.02 9.04 5.93 4.60 4.71 218 129 22

PSMA15 5.30 2.52 3.65 1.97 4.20 3.90 202 122 36

PSMA16 6.00 3.03 6.23 3.80 4.60 4.20 346 238 34

PSMA18 3.70 1.84 5.24 4.84 4.20 4.00 254 197 62

PSMA19 4.00 1.90 5.22 4.68 3.20 3.30 297 119 77

PSMA20 6.00 3.02 7.63 5.52 4.30 4.30 187 68 27

PSMA21 4.80 2.05 7.55 4.51 4.30 3.50 270 209 123

PSMA23 3.00 1.38 6.19 3.89 3.60 4.00 346 153 28

PSMA24 2.50 1.25 8.20 4.22 3.40 3.10 364 210 27

PSMA26 3.70 1.96 2.86 3.66 3.60 3.40 67 174 70

PSMA27 3.00 1.65 6.54 5.22 3.70 3.60 366 288 76

PSMA28 2.90 1.35 3.53 3.11 3.40 3.04 188 97 41

WBC in cells/nL (range 4-10); erythrocytes in cells/pL (range 4.3-6.1). platelets in cells/nL (range 150-440)
