# Supplementary material for: Radiation dosimetry and first therapy results with a 124I/131I-labeled small molecule (MIP-1095) targeting PSMA for prostate cancer therapy
Source: Eur J Nucl Med Mol Imaging. 2014 Feb 28;41(7):1280–92. doi: 10.1007/s00259-014-2713-y (PMC4052014; doi:10.1007/s00259-014-2713-y)
Supplement: Supplementary file 7 — (PDF 41 kb) [file 259_2014_2713_MOESM7_ESM.pdf]

Supplementary table F Biologically Equivalent Dose (BED) Worksheet for I-131 MIP-1095

Computations from I-124 Data in Patients from Heidelberg, Germany

**Assumptions**						
alpha/beta=	**2,6**	Gy	G-function= Lea-Catcheside function for bi-exponential clearance	Kidney mass	299	gm (OLINDA phantom)
T(repair)=	**2,8**	hr	BED = D(cort)*RE	Tp(I-131)	192,48	hr
µ(repair)=	**0,248**	/hr	RE = 1 + G*D(cort)/(alpha/beta)	Lambda_P	0,00360	
y mean density	**1,00**	g/cc				

Patient ID	m(kidney) grams	D(phan) Gy	D(patient) Gy	a1(kid)	a2(kid)	lambda_1(Bio) /hr	lambda_2(Bio) /hr	lambda_1(eff) /hr	lambda_2(eff) /hr	G1,2-function	A(total) GBq
98											
07	314,8	2,9	2,8	0,018228	0,011782	0,40151	0,028707	0,4051	0,0323	0,1289	7,2
04	437,19	13,9	9,5	0,066277		0,009539		0,0131	0,0036	0,0504	6,0
05	258,56	1,0	1,1	0,004697	0,003771	0,468341	0,033809	0,4719	0,0374	0,1418	3,5
02	305,27	2,0	2,0	0	0,013537	0,004525	0,009568	0,0081	0,0132	0,0505	3,4
01	398,61	4,6	3,4	0	0,02271	0,202355	0,009089	0,2060	0,0127	0,0488	5,0
03	665,62	7,7	3,5	0,040268		0,012804		0,0164	0,0036	0,0622	6,7
10	376,82	4,2	3,3	0,02787		0,01079		0,0144	0,0036	0,0549	4,7
09	530,78	10,7	6,0	0,013469	0,026378	0	0,044828	0,0036	0,0484	0,0190	5,4
11	520,46	12,0	6,9	0,045709		0,006465		0,0101	0,0036	0,0391	6,0
06	460,42	11,0	7,2	0,010385	0,012381	0,036299	0	0,0399	0,0036	0,0163	5,9
16	593,66	10,0	5,0	0,055095		0,011306		0,0149	0,0036	0,0568	6,0
08	682,84	17,1	7,5	0,019044	0,00944	0	0,09571	0,0036	0,0993	0,0148	6,8
15	692,01	7,2	3,1	0,041858		0,010936		0,0145	0,0036	0,0555	5,3
13	757,4	11,4	4,5	0,036088	0	0,005753	0,000341	0,0094	0,0039	0,0364	6,3
99										NA	

Patient ID	D(cort) Gy	RE@D(cort)	BED Gy	D(cort)/A Gy/GBq	# Fractions 1 Activity for 37 Gy BED GBq	# Fractions 1 Activity for 44 Gy BED GBq	# Fractions 2 Activity for 37 Gy BED GBq	# Fractions 2 Activity for 44 Gy BED GBq	# Fractions 3 Activity for 37 Gy BED GBq	# Fractions 3 Activity for 44 Gy BED GBq
07	3,97	1,20	4,75	0,55	34,6	38,8	42,5	48,2	47,0	53,7
04	13,54	1,26	17,10	2,26	11,1	12,6	12,8	14,7	13,7	15,8
05	1,60	1,09	1,73	0,46	40,4	45,3	50,0	56,6	55,6	63,3
02	2,80	1,05	2,95	0,82	30,3	34,5	35,1	40,4	37,5	43,4
01	4,91	1,09	5,37	0,98	25,6	29,1	29,6	34,1	31,5	36,6
03	4,94	1,12	5,53	0,74	32,0	36,3	37,6	43,2	40,5	46,8
10	4,71	1,10	5,18	1,00	24,4	27,7	28,4	32,6	30,4	35,2
09	8,60	1,06	9,15	1,59	19,0	22,0	20,7	24,2	21,4	25,2
11	9,85	1,15	11,31	1,64	16,1	18,4	18,4	21,2	19,4	22,6
06	10,24	1,06	10,89	1,73	17,9	20,7	19,3	22,6	19,9	23,4
16	7,21	1,16	8,35	1,20	20,1	22,9	23,5	27,0	25,2	29,2
08	10,68	1,06	11,32	1,57	20,0	23,2	21,5	25,2	22,1	26,0
15	4,42	1,09	4,83	0,83	29,2	33,2	34,1	39,2	36,5	42,2
13	6,43	1,09	7,01	1,02	26,3	30,1	29,9	34,6	31,5	36,7
				Mean	24,8	28,2	28,8	33,1	30,9	35,7
				Min	11,1	12,6	12,8	14,7	13,7	15,8
				Max	40,4	45,3	50,0	56,6	55,6	63,3
				SD	8,0	8,9	10,3	11,6	11,7	13,2

Patient ID	# Fractions 1 BED (Gy) 37 D(cort) Gy	# Fractions 1 BED (Gy) 44 D(cort) Gy	# Fractions 2 BED (Gy) 37 D(cort) Gy	# Fractions 2 BED (Gy) 44 D(cort) Gy	# Fractions 3 BED (Gy) 37 D(cort) Gy	# Fractions 3 BED (Gy) 44 D(cort) Gy
07	19,04	21,37	23,41	26,54	25,91	29,56
04	24,94	28,38	28,90	33,27	30,85	35,74
05	18,44	20,68	22,81	25,82	25,33	28,86
02	24,93	28,37	28,89	33,26	30,84	35,73
01	25,14	28,63	29,07	33,48	30,99	35,93
03	23,64	26,81	27,78	31,86	29,88	34,51
10	24,41	27,74	28,45	32,70	30,46	35,25
09	30,28	35,02	33,01	38,56	34,15	40,08
11	26,47	30,25	30,16	34,86	31,90	37,10
06	30,98	35,91	33,48	39,19	34,51	40,56
16	24,20	27,49	28,27	32,48	30,31	35,05
08	31,40	36,46	33,77	39,56	34,72	40,84
15	24,35	27,67	28,40	32,64	30,42	35,19
13	26,88	30,75	30,49	35,28	32,17	37,45

G-function computation (4 components & denominator)

Patient ID	N-term1	N-term2	N-term3	N-term4	Denom	G1,2-function	term1	term2
98								
07	0,001257	0,001505	0,003509	0,015356	0,167837	0,128856	10,089	0,1982
04	1,282658	0,000000	0,000000	0,000000	25,443469	0,050412	25,787	0,0776
05	0,000065	0,000097	0,000244	0,001334	0,012267	0,141843	9,165	0,2182
02	0,000000	0,000000	0,000000	0,053386	1,056770	0,050518	25,734	0,0777
01	0,000000	0,000000	0,000000	0,156208	3,202977	0,048770	26,656	0,0750
03	0,374554	0,000000	0,000000	0,000000	6,025607	0,062160	20,914	0,0956
10	0,206102	0,000000	0,000000	0,000000	3,750851	0,054948	23,659	0,0845
09	0,200667	0,054390	0,046151	0,048551	18,367060	0,019043	68,268	0,0293
11	0,805909	0,000000	0,000000	0,000000	20,622609	0,039079	33,266	0,0601
06	0,009405	0,020569	0,023543	0,169557	13,683260	0,016303	79,741	0,0251
16	0,776029	0,000000	0,000000	0,000000	13,660932	0,056806	22,885	0,0874
08	0,401163	0,013914	0,010074	0,002587	28,992904	0,014753	88,116	0,0227
15	0,459980	0,000000	0,000000	0,000000	8,291723	0,055475	23,434	0,0853
13	0,542091	0,000000	0,000000	0,000000	14,886377	0,036415	35,699	0,0560
99								
						0,055384		

Key to columns/variables

Patient ID	Patient ID number
m(kidney)	patient's total kidney mass from PET/CT (grams, assumes kidney density is 1.0 g/cc)
D(phan)	Kidney radiation dose from OLINDA for total administered activity (Gy)
D(patient)	Kidney radiation dose adjusted for patient-spcific kidney mass (Gy)
a1(kid)	Coefficient #1 of biexponential function for activity in kidney (at t=0; from biological modeling of kidney TAC)
a2(kid)	Coefficient #2 of biexponential function for activity in kidney (at t=0; from biological modeling of kidney TAC)
lambda_1(Bio)	Biological removal rate for component #1 of kidney biomodel (/hr)
lambda_2(Bio)	Biological removal rate for component #2 of kidney biomodel (/hr)
lambda_1(eff)	Effective removal rate for component #1, including I-131 decay
lambda_2(eff)	Effective removal rate for component #2, including I-131 decay
G1,2-function	Lea-Catcheside function for bi-exponential clearance curve for kidney (computed in cells G35-G48)
A(total)	Total therapy administered activity (GBq)
D(cort)	Radiation dose to renal cortex assuming 70% of total renal volume is cortical.
RE@D(cort)	Relative effectiveness function computed for the total renal/cortical radiation dose (valid only for the patient's IA)
BED	Biologically Equivalent Dose for the diven patient and his therapeutic cortical radiation dose
D(cort)/A	Radiation dose to renal cortex per unit administered activity (Gy/GBq)
Activity for X Gy BED	This is the activity needed to deliver a BED of X Gy to the renal cortex for a given patient, Computed for 37 & 44 Gy BED, in 1 fraction
Activity for 2 fractions	This computes the total injected activity that must be split between 2 fractions to achieve 37 & 44 Gy BED
Activity for 3 fractions	This computes the total injected activity that must be split between 3 fractions to achieve 37 & 44 Gy BED
